# Engineered MoS_2_ Nanoplatforms for Drug-Enhanced Cancer Phototherapy: From Design Strategies to Translational Opportunities

**DOI:** 10.3390/nano16080445

**Published:** 2026-04-08

**Authors:** Catarina Tavares, Maria Carolina Dias, Bruno Freitas, Fernão D. Magalhães, Artur M. Pinto

**Affiliations:** 1 LEPABE—Laboratory for Process Engineering, Environment, Biotechnology and Energy, ALiCE—Associate Laboratory in Chemical Engineering, Faculty of Engineering, Universidade do Porto, Rua Dr. Roberto Frias, 4200-465 Porto, Portugal; catarina.gmt@icloud.com (C.T.); fdmagalh@fe.up.pt (F.D.M.); 2 i3S—Instituto de Investigação e Inovação em Saúde, Universidade do Porto, Rua Alfredo Allen, 208, 4200-135 Porto, Portugal; 3 INEB—Instituto de Engenharia Biomédica, Universidade do Porto, Rua Alfredo Allen, 208, 4200-135 Porto, Portugal; 4 IPATIMUP—Instituto de Patologia e Imunologia Molecular da Universidade do Porto, Rua Alfredo Allen, 208, 4200-135 Porto, Portugal

**Keywords:** molybdenum disulfide (MoS_2_), photothermal therapy (PTT), photodynamic therapy (PDT), biocompatibility, drug delivery, cancer treatment

## Abstract

Cancer remains a major global health challenge, and the limitations of conventional therapies have intensified interest in treatment strategies that combine improved selectivity with reduced systemic toxicity. Photothermal therapy and photodynamic therapy have emerged as minimally invasive approaches capable of achieving spatiotemporally controlled tumour ablation. In this context, molybdenum disulfide (MoS_2_), a transition metal dichalcogenide with strong near-infrared absorption, high photothermal conversion efficiency, and versatile surface chemistry, has gained increasing attention as a multifunctional platform for drug delivery and light-triggered cancer therapy. This review examines recent advances in engineered MoS_2_ nanoplatforms for drug-enhanced cancer phototherapy, with emphasis on how surface design and therapeutic cargoes mechanistically amplify light-triggered tumour killing. Approaches such as polymer coatings, biomimetic membranes, targeting ligands, chemotherapeutic agents, nucleic acids, and photosensitisers have been explored to improve colloidal stability, tumour targeting, immune evasion, and stimulus-responsive drug release, while also adding complementary cytotoxic pathways such as chemotherapy, ROS generation, or gene silencing. Available in vitro and in vivo studies indicate that these systems generally exhibit favourable short-term biocompatibility under the tested conditions and can produce significant antitumour effects following irradiation. The review also discusses key biological barriers and translational challenges, including biodistribution, long-term safety, reproducibility, and regulatory considerations, highlighting opportunities for the development of clinically viable MoS_2_-based phototherapeutic platforms.

## 1. Introduction

Cancer refers to the uncontrolled proliferation of genetically altered cells that can invade surrounding tissues and, in many cases, spread to distant sites through metastasis [1]. The development of this disease is related to genetic mutations, generally caused by external factors, including tobacco use, obesity, exposure to ionising radiation, and oncogenic infections such as human papillomavirus (HPV) and hepatitis viruses [2]. Cancer currently represents a major global health burden, with around 22 million new cases and 9.6 million cancer-related deaths registered in 2022, underscoring the persistent need for therapies that are both effective and less damaging to normal tissues [3].

Surgery, radiotherapy, chemotherapy, hormonal therapy, targeted therapy, and immunotherapy, often used in combination for improved efficacy, remain the clinical backbone of cancer management, but their outcomes are often limited by incomplete tumour selectivity, dose-limiting toxicity, acquired resistance, recurrence, and reduced efficacy in advanced or disseminated disease [4]. These limitations have intensified interest in locoregional and stimulus-responsive strategies capable of confining treatment to the tumour site while preserving surrounding tissues [5,6].

**Figure 1:**
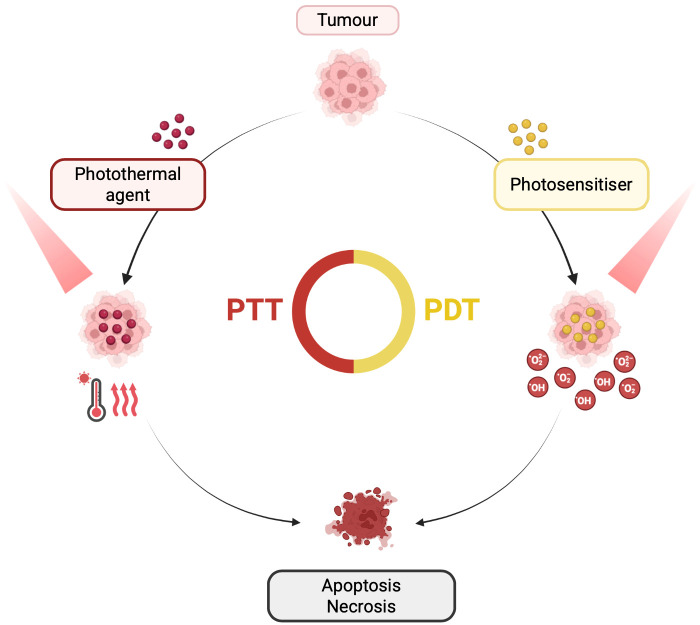
Schematic representation of PTT and PDT. Upon NIR irradiation, photothermal agents convert absorbed light into heat, producing local hyperthermia that can damage or ablate tumour tissue, whereas photosensitisers generate ROS, which induce oxidative stress and cytotoxicity. As illustrated, both mechanisms converge on tumour cell death, highlighting the complementary therapeutic principles of PTT and PDT and their shared end-point of tumour destruction. Created with Biorender.com. Abbreviations: NIR: near-infrared; PDT, photodynamic therapy; PTT, photothermal therapy; ROS: reactive oxygen species.

Phototherapy entered the cancer treatment scene as a minimally invasive alternative with increasing popularity due to its high treatment efficacy and strong spatiotemporal control that results in limited toxicity to surrounding tissues [7,8]. The two most established phototherapeutic modalities are photothermal (PTT) and photodynamic (PDT) therapies, both relying on the interaction between light and photosensitive agents to eliminate cancer cells, with synergistic effects reported when used in combination (Figure 1) [9]. Lasers remain the dominant light source due to their monochromaticity, collimation, and high power density, yet light-emitting diodes (LEDs) are drawing increasing attention for their low cost, portability, scalability over larger illumination areas, and compatibility with metronomic or outpatient workflows [10]. Preclinical and device-development studies have shown that battery-operated or fibre-coupled LED platforms can achieve PDT performance comparable to laser systems in specific settings, while organic light-emitting diode (OLED)-based approaches are now being explored for low-intensity, conformable irradiation [11,12,13].

PDT relies on preferential photosensitiser (PS) accumulation in the tumour (and its vasculature) and subsequent light activation, which in the presence of oxygen (O_2_) generates reactive oxygen species (ROS) that kill cancer cells [14,15,16]. Beyond direct tumour cell killing, PDT can damage tumour vasculature and trigger apoptosis, necrosis, autophagy and immunogenic cell death [17,18]. Upon irradiation, the excited PS can undergo Type I electron-transfer reactions, producing species such as superoxide (O_2_•^−^), hydrogen peroxide (H_2_O_2_), and hydroxyl radicals (•OH), and/or Type II energy-transfer reactions that yield singlet oxygen (^1^O_2_) [19,20,21]. PDT typically operates within a therapeutic window of 600–800 nm, for higher efficiency. Nevertheless, some common PSs absorb outside this range, for instance, protoporphyrin IX shows a stronger absorption band near 400 nm (blue light) [14,15,22]. Despite its promise, PDT can be limited by poor PS water solubility, suboptimal biodistribution, and its degradation in physiological media, as well as the hypoxic tumoural environment and ROS-scavenging defence systems [23,24]. Current design strategies therefore focus on mitochondrial targeting, Type I photosensitisers for hypoxic tumours, oxygen-generating or oxygen-carrying nanoplatforms, and combinations that convert PDT-induced local damage into broader immune activation [25,26,27,28]. Ming et al. [8] explored strategies to enhance ROS generation, showing that increasing excitation-light penetration and improving photoinduced electron and energy transfer can boost ROS production.

Using similar principles, PTT hinges on near-infrared (NIR) light (700–1100 nm) irradiation of photothermal agents (PTAs) to convert light energy into heat, producing local hyperthermia that can directly ablate tumour cells or sensitise them to chemotherapy, immunotherapy, gene therapy, or catalytic therapies, allowing its application in areas where surgery may be challenging [5,29,30]. Most reported systems operate in the NIR-I window, but NIR-II excitation is gaining momentum owing to its deeper tissue penetration, lower scattering, and improved support for image-guided treatment [31,32]. Conventional PTT employs rapid temperature increases exceeding 50 °C to cause cell necrosis, which can be damaging to nearby tissues and destroy the surrounding vasculature, hindering the delivery of therapeutic agents to deep tumour locations [33]. To address this limitation, mild temperature PTT ranging between 42 and 45 °C has been applied to achieve local hyperthermia rather than traditional tumour ablation [34]. Mild PTT can reduce collateral injury while disrupting tumour metabolism, perfusion, DNA-repair capacity, and immune privilege [35,36]. The caveat is that tumours may counter mild heating through heat-shock-protein-driven thermotolerance, which explains the growing interest in nanoplatforms that combine PTT with adenosine triphosphate (ATP) depletion, heat shock protein (HSP) suppression, ROS amplification, or second-mode therapies [37,38].

In order to further reduce damage to healthy tissue, hyperthermia can be achieved using less total light energy by tuning adequate nanoscale materials to behave as PTAs, enhancing the selectivity of PTT within the target tumour tissue [39]. Within this landscape, 2D nanomaterials (2DnMats) have become attractive because of their ultrathin geometry, high surface-to-volume ratio, accessible active sites, and rich surface chemistry support high cargo loading, multivalent functionalisation, and multimodal imaging-therapy integration [40,41,42,43]. The introduction of 2DnMat into cancer research began with graphene-based materials (GBMs) [44]. However, other 2D material families later gained recognition, including transition metal dichalcogenides (TMDs), transition metal oxides (TMOs), transition metal carbides, nitrides and carbonitrides (MXenes), black phosphorus (BP) nanosheets, layered double hydroxides (LDHs), and others. These systems have been explored as drug carriers, PTAs, photosensitiser supports, gene-delivery vehicles, and photoimmunotherapy scaffolds [45,46,47,48,49,50]. At the same time, their biological behaviour cannot be separated from the nano–bio interface. Size, thickness, charge, defect density, phase composition, coating, and degradability influence protein corona formation, uptake, trafficking, clearance, immune reactivity, and ultimately both efficacy and safety [51]. These materials can also undergo surface modification to enable targeted delivery, focusing on specific receptors expressed by cancer cells and thereby increasing therapy selectivity, making them ideal drug delivery systems (DDSs) [52,53].

Molybdenum disulfide (MoS_2_) is a promising TMD, often preferred to GBM, BP, or MXenes for its controlled tunability during preparation, and its strong NIR absorption, photothermal responsiveness, large surface area, and ability to bind drugs, nucleic acids, proteins, or targeting ligands through covalent and non-covalent strategies make it especially suitable for combination therapy [30,41,54,55,56]. In its natural form, MoS_2_ has a triangular prismatic structure, taking the form of a side rhombus after top-down or bottom-up synthesis. It can assume three different crystal structures, namely hexagonal 2H, octahedral 1T, and rhombohedral 3R phases. In a monolayer, the sulphur (S) atoms are located in two different planes that entrap molybdenum (Mo) atoms in a sandwich conformation, through covalent bonding. The weak interlayer van der Waals forces provide easy exfoliation [30,52,57]. MoS_2_’s unique properties, including distinctive band gap structure, high carrier mobility, high absorbance in the near-infrared region, remarkable magnetic features, mechanical strength, and high surface-to-volume ratio, make it suitable for biological, optics, and electronics applications [52,57]. MoS_2_ presents other advantages for biomedical applications, such as an extensive range of raw material sources, low production cost, simple preparation, good chemical stability and versatility, and high photothermal conversion efficiency [52].

**Figure 2:**
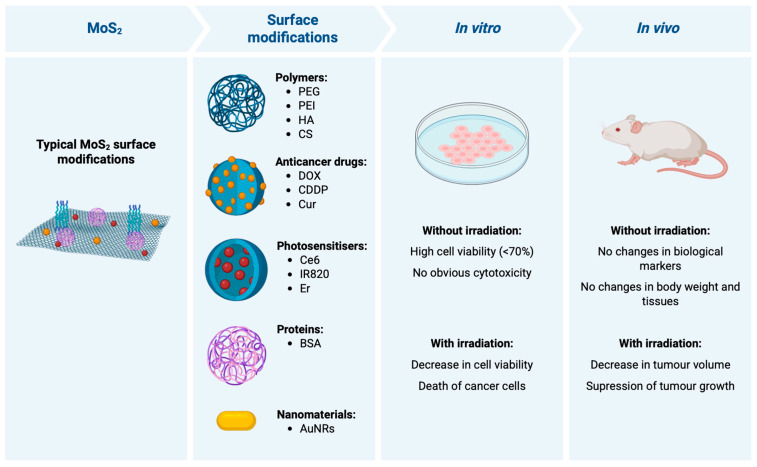
Schematic overview of common surface modification strategies and representative biological outcomes of MoS_2_-based nanoplatforms for cancer therapy. MoS_2_ nanosheets are frequently functionalised with polymers (e.g., PEG, PEI, HA, CS), anticancer drugs (e.g., DOX, CDDP, Cur), photosensitisers (e.g., Ce6, IR820), proteins (e.g., BSA), and other nanomaterials (e.g., Au nanorods) to improve colloidal stability, biocompatibility, targeting, and therapeutic efficacy. In in vitro models, these systems typically show low dark cytotoxicity and high cell viability in the absence of irradiation, whereas light exposure leads to reduced cancer cell viability and increased tumour cell death. In in vivo models, they generally exhibit limited systemic toxicity without irradiation, with no major changes in body weight, tissues, or key biological markers, while irradiation results in marked tumour growth inhibition or suppression. Created with Biorender.com. Abbreviations: AuNRs: gold nanorods; BSA: bovine serum albumin; Ce6: chlorin e6; CDDP: cisplatin; CS: chitosan; Cur: curcumin; DOX: doxorubicin; Er: erlotinib; HA: hyaluronic acid; IR820: new indocyanine green; MoS_2_: molybdenum disulfide; PEG: polyethylene glycol; PEI: polyethylenimine.

MoS_2_-based materials developed for cancer treatment typically present surface modifications that enhance therapeutic properties, including functionalisation with polymers, anticancer drugs, PS, proteins, among others. These modified nanomaterials have been tested in in vitro and in vivo assays, in the presence and absence of light irradiation, to evaluate their biocompatibility and anticancer effects (Figure 2).

Several recent reviews have already summarised MoS_2_ in broad cancer diagnosis and therapy, multimodal theranostics, or general anticancer drug-delivery contexts [29,30,52,54,55,56]. The distinctive focus of the present review is narrower and more mechanism-oriented. Rather than surveying MoS_2_ across all biomedical applications, this review specifically examines engineered nanoplatforms in which surface modification and therapeutic cargo integration are used to enhance phototherapy. Particular emphasis is placed on (i) how different functionalisation strategies govern colloidal stability, targeting, immune interaction, and triggered release; (ii) how chemo-photothermal, photothermal-photodynamic, and more recent gene- or radical-assisted combinations differ mechanistically; and (iii) how these design choices influence short-term biocompatibility, unresolved long-term biosafety, and translational feasibility. In this way, the review is intended to complement broader MoS_2_ reviews by providing a focused framework for drug-enhanced cancer phototherapy and its path toward clinical translation.

## 2. MoS_2_ Surface Functionalisation and Drug Conjugation Strategies

As a transition metal, Mo has multiple valence states that allow surface modification and doping with other elements. Unlike some inorganic nanomaterials, such as gold-based platforms, MoS_2_ is composed of elements naturally present in the human body, contributing to the good biocompatibility demonstrated in numerous studies [30]. The versatility of MoS_2_ allows bonding with polymers, smaller organic molecules, and biological molecules through covalent or non-covalent interactions, developing new platforms with great potential for drug delivery and cancer phototherapy [29].

Chemical functionalisation establishes new covalent bonds between molecules and the S or Mo atoms, facilitated by defects and vacancies in the crystal lattice [52]. Meanwhile, non-covalent strategies can be employed to modify MoS_2_-based nanomaterials through hydrophobic interactions, hydrogen bonding, van der Waals forces, π−π stacking forces and electrostatic adsorption, enabling binding of nucleic acids, proteins, and other macromolecules. Additionally, it is possible to apply polymer coatings via physical interaction with biocompatible molecules, such as polyethylene glycol (PEG) [29]. Several studies have investigated MoS_2_ surface modification for enhancing PTT performance (Table 1).

Doxorubicin (DOX), one of the most widely used chemotherapeutic agents in cancer treatment owing to its broad-spectrum activity [68], is the drug most frequently conjugated with MoS_2_ in these studies. Liu et al. [58] reported a DOX@Biotin-BSA-PEI-LA-MoS_2_-LA-PEG platform prepared by adsorption of lipoic acid-polyethyleneimine (LA-PEI) onto MoS_2_, followed by formation of PEI-LA-MoS_2_-LA-PEG, adsorption of biotin-bovine serum albumin (BSA) obtained by 1-ethyl-3-(3-dimethylaminopropyl)carbodiimide (EDC)/N-hydroxysuccinimide (NHS) coupling, and subsequent DOX loading. This formulation showed improved cellular uptake through biotin-mediated targeting. At a drug:material ratio of 2.5:1 and pH 5.5, the system exhibited an encapsulation efficiency (EE) of 23.35% and a drug loading capacity (DL) of 34.43%. After 72 h, DOX release was higher at pH 5.5 than at pH 7.0 (30% versus 15%) and increased further under 808 nm laser irradiation (1.5 W·cm^−2^, 30 min), reaching 42% and 23%, respectively.

Dong et al. [59] prepared DOX@MoS_2_-PEI-HA by first forming MoS_2_-PEI through electrostatic interaction and then coupling PEI amine groups with (HA) carboxyl groups, followed by DOX loading. In addition to good photostability, this system improved biocompatibility, enabled CD44 targeting, and showed enzyme-responsive release associated with HA degradation. The DL reached 33.6% at pH 8.0. Over 6 h, release increased from 11.2% at pH 7.4 to 25.5% at pH 5.0, while hyaluronidase (HAase) further enhanced release to 23.2% and 41.6%, respectively. Irradiation with an 808 nm laser (0.6 W·cm^−2^, 10 min) increased release at pH 5.0 to 35.8%, and the combination of irradiation and HAase produced the highest value, 77.4% at pH 5.0.

Additional DOX-loaded systems further illustrate how the surface layer modulates stability, biological behaviour, and release kinetics. Chitosan (CS)-functionalised MoS_2_ (MoS_2_-CS), obtained during exfoliation, displayed high physiological stability and improved biocompatibility, with a DL of 32% at pH 8.0. In this case, DOX release remained low without irradiation (6% after 1 h at pH 8.0) but increased to 12.4% after 808 nm irradiation (0.8 W·cm^−2^, 10 min) [61]. Red blood cell (RBC) membrane-coated MoS_2_ (MoS_2_-RBC) combined improved stability in water with immune-evasive properties, including reduced macrophage phagocytosis and protein adsorption. This formulation showed a DL of 98.98% at a drug:material ratio of 2:1 and pH 7.4, whereas DOX release after 4 h remained modest, at approximately 14.5% at pH 5.5 and 7% at pH 7.4; under 808 nm irradiation (2 W·cm^−2^), these values increased only slightly to approximately 15% and 8%, respectively [60]. A more complex architecture, MoS_2_-AuNRs-HAP-PDA, was generated through in situ growth of gold nanorods (AuNRs) on MoS_2_ followed by self-assembly of hydroxyapatite (HAP) and polydopamine (PDA). DOX was loaded through PDA adhesion and electrostatic interaction with HAP, yielding an EE of 80% at a drug:material ratio of 0.075:1 and pH 7.4. This system showed increased stability in aqueous solutions and enhanced PTT efficacy, while DOX release after 10 h increased from approximately 12% at pH 7.4 to 31.66% at pH 4.5, and further to approximately 15% and 48.85%, respectively, under 808 nm irradiation (4 W·cm^−2^) [63]. Collectively, these studies indicate that DOX release from MoS_2_-based platforms generally increases under acidic conditions and is often further enhanced by NIR irradiation, consistent with pH-/NIR-responsive release behaviour reported for MoS_2_ nanocarriers. In MoS_2_-PEI-HA, this response is further strengthened by hyaluronidase-mediated degradation of the HA shell, which accelerates DOX release, while mild 808 nm irradiation also promotes release in the acidic tumour environment [59].

Taken together, these DOX-based studies illustrate differences in design priorities rather than simple repetition of the same strategy. The Biotin-BSA-PEI-LA-MoS_2_-LA-PEG platform [58] mainly focused on improving colloidal stability and active cellular uptake through biotin-mediated targeting, while preserving pH- and NIR-responsive release. MoS_2_-PEI-HA [59] advanced this concept by introducing a biologically relevant HA shell that improved biocompatibility, enabled CD44-mediated targeting, and added enzyme responsiveness through HA degradation, thereby producing a more selective and more strongly triggered release profile. By contrast, MoS_2_-RBC [60] prioritised prolonged circulation and immune evasion through membrane camouflage, but showed only modest irradiation-enhanced release, suggesting that stealth behaviour does not necessarily maximise on-demand drug liberation. MoS_2_-CS [61] represented a simpler stabilisation strategy that improved biocompatibility and physiological stability with comparatively moderate NIR-triggered release. Finally, the more complex MoS_2_-AuNRs-HAP-PDA hybrid [63] shifted the design emphasis toward photothermal amplification and stronger acid/NIR-responsive release by integrating Au nanorods and additional functional layers. Overall, the main distinction between these studies lies in whether the design was primarily intended to improve tumour cell targeting, enzyme responsiveness, immune evasion, formulation simplicity, or photothermal performance. Although the FA-MoS_2_-peptide [66] also contains DOX, it should be viewed separately from these DOX-only systems because its key advance lies in FA-mediated targeted co-delivery of DOX and anti-Gal-1 siRNA, together with lysosomal escape and gene-silencing capability, representing a transition toward multifunctional chemo-gene-photothermal nanoplatforms.

MoS_2_ has also been explored as a carrier for other chemotherapeutic agents. In the case of cisplatin (CDDP), PDA was first introduced onto MoS_2_ by self-polymerisation of dopamine, after which poly(2-methacryloyloxyethyl phosphorylcholine-itaconic acid) [poly(MPC-IA)] was grown by surface-initiated single electron transfer living radical polymerisation (SET-LRP). CDDP was then coordinated to carboxyl groups on the copolymer-coated surface. The resulting MoS_2_-PDA-poly(MPC-IA) nanomaterial exhibited improved dispersibility in phosphate-buffered saline (PBS) and a DL of 55.26% at a drug:material ratio of 0.27:1 and pH 7.4. Release after 48 h was markedly higher at pH 5.5 than at pH 7.4 (49% versus 15%) [62].

In addition to chemotherapeutic drugs, MoS_2_ has been used to load photosensitisers and other therapeutic agents. PEG-modified MoS_2_ loaded chlorin e6 (Ce6) by physical adsorption, reaching a DL of 30% at a drug:material ratio of 2.5:1 and pH 6.0. This formulation also showed stability in physiological solutions, enhanced cellular uptake, and ROS generation [7]. Likewise, PEI-functionalised MoS_2_ loaded IR820 through electrostatic interaction, showing a DL of 9.17% at a drug:material ratio of 1:3 and pH 7.4, while retaining ROS-generating capability [64]. Chen et al. [65] further reported an MoS_2_-PEG-Biotin system, prepared through thiol-mediated grafting of PEG-biotin onto MoS_2_, for the adsorption of curcumin (Cur) and erlotinib (Er). The DL values were 22.3% for Cur and 10.1% for Er. After 2 h, release reached 7.4% for Cur and 5.2% for Er, and these values increased to 16.8% and 12%, respectively, after 10 min of 808 nm irradiation (0.6 W·cm^−2^). This platform also showed increased cellular uptake through biotin-mediated targeting.

More recent studies have further expanded the functional scope of MoS_2_-based platforms beyond conventional small-molecule loading. Chibh et al. [66] described an FA-MoS_2_-peptide nanosystem obtained by exfoliating bulk MoS_2_ in aqueous self-assembled peptide nanosheets, followed by folic acid (FA) conjugation and sequential loading of DOX and anti-galectin-1 (Gal-1) small interfering ribonucleic acid (siRNA). This formulation combined FA-mediated targeting with protection of the siRNA cargo against ribonuclease degradation, while DOX release increased from approximately 63% to 90% after 808 nm irradiation. Yin et al. [67] further reported an MoS_2_-lauric acid (LAU) platform loaded with 2,2′-Azobis [2-(2-imidazolin-2-yl)propane] dihydrochloride (AIPH), in which the surface coating improved colloidal stability, reduced premature leakage, and enabled thermo-responsive release. In this case, drug loading reached approximately 70%, and laser irradiation increased release from 2% to 17%. This system was built to support ROS generation under hypoxic conditions via thermal-induced decomposition of the free radical initiator AIPH.

Compared with the earlier DOX-based systems, these later platforms do not merely refine drug loading or trigger control, but broaden the therapeutic concept itself. The CDDP-loaded MoS_2_-PDA-poly(MPC-IA) system mainly advanced polymer-mediated dispersibility and pH-responsive coordination release, whereas Ce6- and IR820-loaded formulations shifted the design objective from chemo-photothermal therapy toward photothermal-photodynamic action through ROS generation. The Cur/Er@MoS_2_-PEG-Biotin platform further extended this strategy to the co-delivery of two small-molecule drugs with ligand-mediated uptake. FA-MoS_2_-peptide and MoS_2_-LAU represent a further increase in functional complexity. The former integrates targeted co-delivery of DOX and siRNA with lysosomal escape, whereas the latter is designed for thermo-triggered oxygen-independent radical generation under hypoxic conditions. Thus, the progression across these studies is best understood not simply as a change in cargo, but as a shift from improved delivery toward increasingly multimodal and mechanism-diverse therapeutic platforms.

Overall, these studies highlight that surface engineering is a key determinant of the therapeutic performance of MoS_2_-based nanoplatforms. By selecting appropriate coatings, ligands, or hybrid structures, it is possible to improve colloidal stability, modulate drug loading and release behaviour, and enhance biological interactions such as cellular uptake, targeting, or immune evasion, while preserving the photothermal functionality of MoS_2_. Collectively, these findings indicate that rational surface modification is essential for optimising MoS_2_ as a multifunctional platform for combined cancer therapy.

## 3. In Vitro Biocompatibility Studies

Biocompatibility testing of materials or drugs is an essential step prior to their application in the biomedical field, ensuring their integration with biological systems while minimising the risk of adverse effects. Surface modifications, which involve the alteration of the material’s properties, can be implemented to enhance biocompatibility [29]. According to Murali et al. [69], the degree of exfoliation can have an impact on the cytotoxicity of MoS_2_. Highly exfoliated MoS_2_ exhibits increased cytotoxicity compared to the non-exfoliated form, attributed to its larger surface area and higher number of active edges. However, it was mentioned that dispersions of aggregated MoS_2_ present higher levels of cytotoxicity than well-exfoliated stable dispersions. Other parameters, including composition, molecular structure, and size, can also influence the cytotoxicity of nanomaterials [70].

In vitro studies typically provide relevant information about the biological interactions occurring at cellular or multicellular levels. Table 2 compiles studies on the in vitro biocompatibility of MoS_2_-based materials and their drug conjugates.

**Figure 3:**
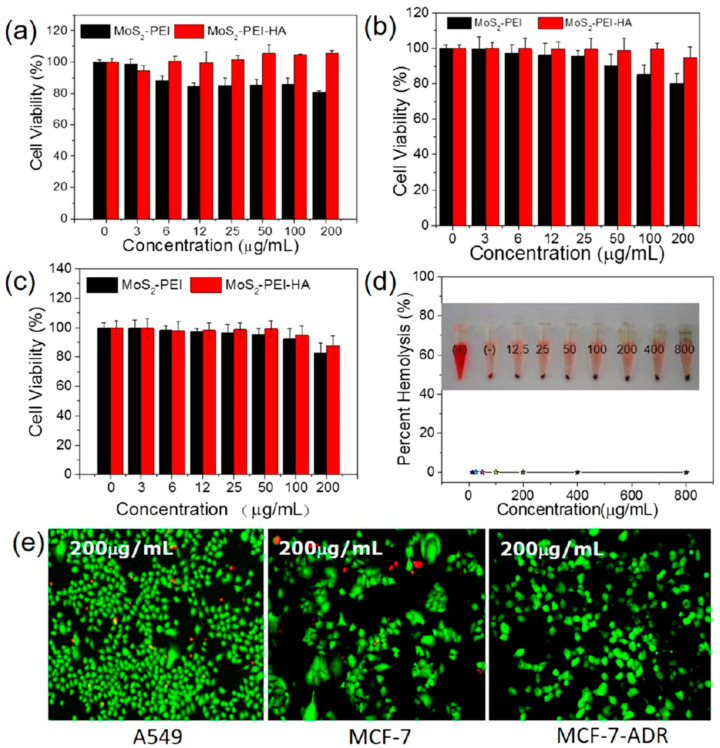
In vitro biocompatibility of MoS_2_-PEI-HA. (**a**–**c**) Cell viability of (**a**) A549, (**b**) MCF-7, and (**c**) MCF-7-ADR cells after treatment with MoS_2_-PEI or MoS_2_-PEI-HA at different concentrations. Across all three cell lines, cell viability remained high over the tested concentration range, and the HA-modified formulation generally showed higher viability than MoS_2_-PEI, indicating that HA coating improves biocompatibility and reduces dark cytotoxicity. (**d**) In vitro haemolysis percentage of red blood cells incubated with MoS_2_-PEI-HA at various concentrations remained negligible even at the highest tested concentration (800 μg·mL^−1^), supporting good blood compatibility. (**e**) Fluorescence live/dead staining of A549, MCF-7, and MCF-7-ADR cells treated with MoS_2_-PEI-HA at 200 μg·mL^−1^, showing predominantly live (green) cells and few dead (red) cells, which further confirms its low intrinsic cytotoxicity. Reprinted with permission from [59]. Copyright 2018 American Chemical Society. Abbreviations: A549: adenocarcinomic human alveolar basal epithelial cell line; HA: hyaluronic acid; MCF-7: human breast adenocarcinoma cell line; MCF-7-ADR: adriamycin-resistant human breast adenocarcinoma cell line; MoS_2_: molybdenum disulfide; PEI: polyethylenimine.

The studies compiled in Table 2 indicate that most MoS_2_-based formulations display favourable in vitro biocompatibility within the concentration ranges investigated. Liu et al. [58] reported that Biotin-BSA-PEI-LA-MoS_2_-LA-PEG (200 nm) maintained 105% viability in HeLa cells after 24 h of incubation at 500 µg·mL^−1^, with cellular internalisation occurring through biotin-mediated endocytosis. Dong et al. [59] found that MoS_2_-PEI-HA (30–50 nm) preserved 105% viability in A549 cells and approximately 95% viability in MCF-7 cells after 24 h at 200 µg·mL^−1^. Cell uptake was CD44-mediated, and haemolysis was negligible at 800 µg·mL^−1^ after 3 h of incubation with RBCs. These findings were confirmed by fluorescence imaging with live/dead staining and are illustrated in Figure 3. Li et al. [60] also observed 101% viability in MCF-7 cells after 24 h of exposure to MoS_2_-RBC (232 nm) at 50 µg·mL^−1^. In addition, this material showed reduced phagocytosis by macrophages, together with reduced BSA adsorption. For MoS_2_-CS (80 nm), Yin et al. [61] reported 85% viability in KB cells and 99% viability in Panc-1 cells after 24 h at 400 µg·mL^−1^, with negligible haemolytic activity at 800 µg·mL^−1^ after 3 h of incubation with RBCs.

A similarly favourable tendency was observed for the remaining formulations. MoS_2_-PDA-poly(MPC-IA) maintained 90% viability in L929 cells after 24 h at 100 µg·mL^−1^ [62]. MoS_2_-PEG yielded 85% viability in 4T1 cells after 12 h at 4 mg·mL^−1^ [7]. For MoS_2_-AuNRs-HAP-PDA (100–200 nm), viability values of 85.32% in EA.hy926 cells and 86.92% in MCF-7 cells were reported after 24 h at 50 µg·mL^−1^ [63]. MoS_2_-PEI (340 nm) showed 91% viability in L929 cells and 98% viability in 4T1 cells after 24 h at 125 µg·mL^−1^ [64]. Chen et al. [65] further illustrated that MoS_2_-PEG-Biotin (120 nm) maintained 90% viability in both HELF and A549 cells after 72 h at 500 µg·mL^−1^. This formulation underwent biotin-mediated uptake and caused negligible haemolysis at 500 µg·mL^−1^ after 2 h of incubation with RBCs.

Two additional studies are consistent with this generally favourable in vitro biocompatibility profile. FA-MoS_2_-peptide maintained approximately 90% viability in L929 cells after 24 h at 2.5 µg·mL^−1^, while folic acid functionalisation increased uptake by approximately 4.2-fold and the nanosystem showed initial lysosomal localisation followed by substantial escape after 12 h [66]. Likewise, MoS_2_-LAU preserved approximately 95% viability in L929 cells at 200 µg·mL^−1^ and caused negligible haemolysis at the same concentration after 2 h of incubation with RBCs [67].

Importantly, the individual studies differ in the specific biological barrier addressed by the surface modification. Biotin-BSA-PEI-LA-MoS_2_-LA-PEG and MoS_2_-PEG-Biotin mainly emphasised ligand-mediated uptake while maintaining high viability, whereas MoS_2_-PEI-HA improved blood compatibility and receptor-mediated internalisation relative to less shielded cationic surfaces. MoS_2_-RBC, by contrast, was distinguished by reduced macrophage phagocytosis and protein adsorption, reflecting a stronger focus on immune evasion. Simpler coatings such as CS, PEG, or poly(MPC-IA) primarily acted as passivating layers that preserved cell compatibility, while FA-MoS_2_-peptide added an intracellular-trafficking advantage through lysosomal escape. Thus, the key difference among these studies is not whether the materials were broadly biocompatible under dark conditions, but which aspect of nano–bio interaction was most improved by the chosen modification.

Taken together, these studies show that the viability of the tested cell lines was not markedly compromised by MoS_2_-based materials under the reported experimental conditions, according to the ISO-10993 definition of cytotoxicity (cell viability below 70% limit) [71]. This favourable profile likely reflects the fact that many of the evaluated formulations incorporated surface coatings such as HA, PEG, CS, proteins, or biomimetic membranes, which can improve colloidal stability, reduce aggregation, and shield the reactive MoS_2_ surface from direct nonspecific interactions with cell membranes. Moreover, in the studies in which blood compatibility was evaluated, haemolysis was consistently negligible, which is consistent with reduced erythrocyte membrane disruption after surface passivation. Where uptake and intracellular localization were assessed, receptor-mediated internalisation or cytoplasmic distribution was generally reported, suggesting that these nanomaterials can be internalised efficiently while remaining below the acute cytotoxicity threshold under the tested dark conditions. Surface coatings are widely used in nanomedicine precisely for their ability to reduce nonspecific protein adsorption, improve colloidal behaviour, and modulate cell interaction and uptake [72,73,74,75,76].

## 4. In Vivo Biocompatibility Studies

In vivo studies are essential for assessing the toxicity and biocompatibility of nanomaterials, as they enable the evaluation of their physical, chemical, and biological interactions within living organisms [77]. In addition to providing a general indication of safety, these studies make it possible to examine toxicokinetic behaviour, including circulation and tissue accumulation after administration, together with potential adverse effects at the organ and systemic levels. Table 3 summarises the currently available in vivo data for MoS_2_-based 2DnMat, including different material formulations evaluated in murine models, using intravenous (IV) or intratumoural (IT) administration, providing an overview of the in vivo safety profile of these materials before considering the individual studies in greater detail.

**Figure 4:**
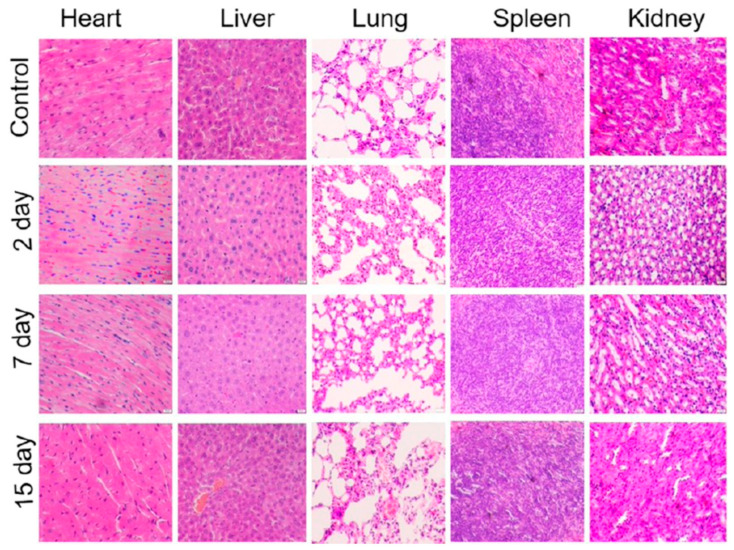
Histological images of H&E-stained sections of the heart, liver, lung, spleen, and kidney collected from mice treated with MoS_2_-PEI-HA and sacrificed at 2, 7, and 15 days after administration. No obvious histopathological alterations are observed in the treated groups compared with the control, and tissue architecture remains preserved across all examined organs and time points. The absence of evident inflammation, necrosis, or structural damage suggests that MoS_2_-PEI-HA does not induce detectable acute or short-term organ toxicity under the tested conditions, supporting its favourable in vivo biocompatibility. Reprinted with permission from [59]. Copyright 2018 American Chemical Society.

Following IV administration of MoS_2_-PEI-HA (10 mg·kg^−1^) in female BALB/c mice, accumulation at 4 h was observed in the liver, spleen, and lungs, as well as the tumour site. Despite this distribution profile, histological analysis revealed no obvious damage or inflammation in the major organs over 15 days, and no significant body-weight loss was detected. Representative haematoxylin and eosin (H&E)-stained sections of organs from mice treated with MoS_2_-PEI-HA are shown in Figure 4 [59]. Similarly, MoS_2_-RBC showed prolonged circulation and accumulated in the liver, spleen, and tumour within 24 h, again without significant changes in body weight [60].

Consistent findings were reported for other formulations. IT administration of MoS_2_-CS (2 mg·kg^−1^) to male BALB/c nude mice resulted in no significant body-weight loss during the 24-day observation period [61]. In 4T1 tumour-bearing BALB/c mice, IV-administered MoS_2_-PEG (6.85 mg·kg^−1^) accumulated in the tumour and caused neither detectable damage nor inflammation in the major organs, while body weight remained unchanged [7]. Likewise, MoS_2_-PEI, administered intravenously at 100 μg·mL^−1^ (100 μL), also accumulated in the tumour and was associated with normal serum biochemistry markers, absence of histological abnormalities in the major organs, and no significant body-weight loss after 14 days [64]. A similar safety profile was observed for MoS_2_-PEG-Biotin in A549 tumour-bearing BALB/c nude female mice, in which accumulation was detected in the liver, spleen, and tumour following IV administration (8 mg·kg^−1^), without evident organ damage or changes in body weight over 21 days [65].

Additional recent studies also support a generally favourable in vivo tolerability profile. Following IV administration, FA-MoS_2_-peptide accumulated in the liver, kidney, lung, spleen, brain, and tumour in male Wistar rats, yet no obvious damage or inflammation was reported in the major organs, and no significant body-weight loss was observed [66]. Similarly, MoS_2_-LAU accumulated in the liver, spleen, and tumour after IV administration in healthy Kunming (KM) mice, while histological analysis showed no evident organ damage or inflammation and body weight remained stable [67].

A more informative comparison of the in vivo studies shows that the reported tolerability profiles are broadly similar, but the underlying biodistribution behaviour differs substantially. HA- or PEG-based systems and PEG-Biotin formulations typically showed tumour accumulation together with liver and spleen deposition, whereas MoS_2_-RBC was distinguished by prolonged circulation, consistent with its membrane-camouflage design. MoS_2_-CS should be interpreted separately, since intratumoural administration bypasses part of the systemic-distribution problem faced by the intravenously injected platforms. The FA-MoS_2_-peptide study further broadened the observed distribution pattern to include kidney, lung, spleen, brain, and tumour, reflecting both the higher functional complexity of the platform and the different biological setting. Accordingly, these studies do not point to a single superior biodistribution strategy, but rather show that formulation and administration route redistribute the trade-off between circulation, tumour access, systemic exposure, and ease of safety interpretation.

Taken together, the available in vivo evidence suggests that MoS_2_-based nanomaterials exhibit good short-term biocompatibility under the evaluated conditions, as reflected by the absence of marked alterations in body weight, serum biochemistry, or tissue morphology. These parameters are commonly used to detect overt acute systemic toxicity, major organ injury, or substantial inflammatory damage after administration. However, this interpretation should remain cautious, since most of the reviewed studies relied on relatively short follow-up periods and routine endpoints, which are better suited to detect short-term intolerance than long-term retention, degradation-related toxicity, or subtle organ-specific effects.

## 5. MoS_2_/Drug Conjugates for Cancer Phototherapy—In Vitro Studies

In vitro phototherapy studies provide an essential framework for evaluating the therapeutic potential of MoS_2_-based nanoplatforms under well-controlled experimental conditions. These experiments enable direct comparison of different surface modifications, drug-conjugated systems, and irradiation protocols, while minimising the biological complexity inherent to in vivo models. As summarised in Table 4, a wide range of MoS_2_-based materials have been investigated in different cancer cell lines, including systems functionalized with polymers, targeting ligands, or biomolecules and combined with chemotherapeutic agents or photosensitisers. The compiled studies also report key experimental parameters that influence treatment outcomes, such as irradiation modality, wavelength, power density, exposure time, incubation period, and nanoparticle concentration.

The available in vitro evidence highlights two main aspects of MoS_2_-based phototherapy platforms. First, MoS_2_ nanomaterials exhibit intrinsic photothermal cytotoxicity upon near-infrared irradiation, which can induce significant reductions in tumour cell viability. Second, therapeutic cargoes frequently enhance this response through several non-mutually exclusive mechanisms. In this review, the term drug-enhanced phototherapy is used broadly to denote MoS_2_ systems in which the loaded therapeutic cargo—whether a chemotherapeutic, photosensitiser, nucleic acid, or radical-generating agent—actively amplifies light-triggered tumour killing by improving tumour-selective accumulation, enabling pH-/enzyme-/NIR-responsive release, overcoming resistance, or adding a complementary cytotoxic modality. Importantly, the synergistic mechanisms differ across the combinations reviewed here. In chemo-PTT systems, which constitute the majority of the studies, MoS_2_-mediated hyperthermia mainly promotes on-demand cargo release and increases tumour cell susceptibility to the co-delivered agent, while the drug provides an orthogonal molecular insult. In PTT/PDT systems, by contrast, synergy arises from the coexistence of heat-mediated injury and photosensitiser-derived reactive oxygen species, which affect partially different intracellular targets. More complex platforms extend this logic further: FA-MoS2-peptide combines PTT with chemotherapy and gene silencing, whereas MoS2-LAU/AIPH couples photothermal heating with thermally triggered oxygen-independent radical generation. Consequently, the label synergistic should be interpreted mechanistically rather than generically, since different combinations do not share the same biological basis or translational rationale.

**Figure 5:**
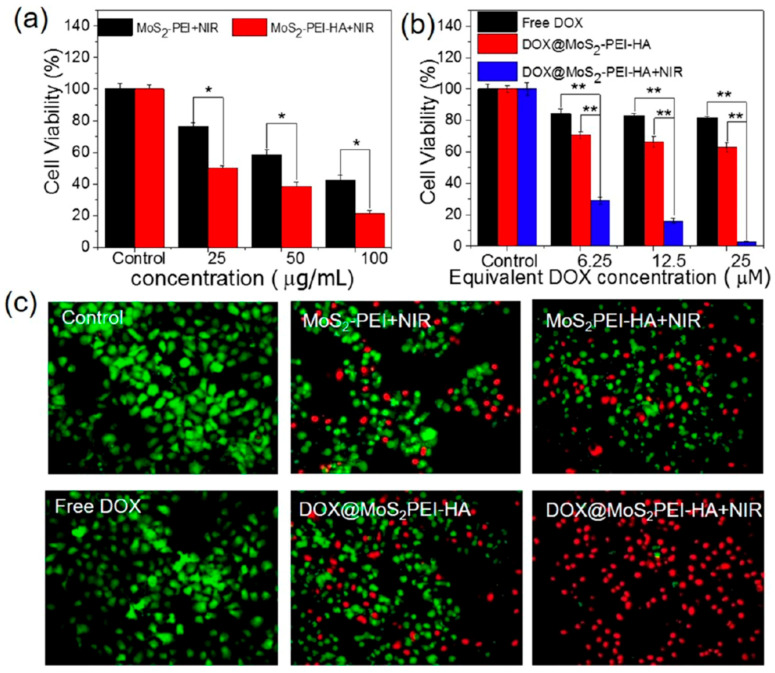
In vitro therapeutic efficacy of MoS_2_-based formulations in MCF-7-ADR cells. (**a**) Cell viability of MCF-7-ADR cells treated with MoS_2_-PEI + NIR or MoS_2_-PEI-HA + NIR after irradiation with an 808 nm laser. MoS_2_-PEI-HA + NIR led to a stronger reduction in cell viability compared to MoS_2_-PEI + NIR at all tested concentrations, indicating that HA modification improves phototherapeutic efficacy. (**b**) Cell viability of MCF-7-ADR cells after treatment with free DOX or DOX@MoS_2_-PEI-HA, with or without irradiation using an 808 nm laser, showed that DOX loading into the MoS_2_-PEI-HA platform enhanced cytotoxicity compared with free DOX alone, while the combination of DOX@MoS_2_-PEI-HA with NIR caused the largest decrease in viability, consistent with a synergistic chemo-photothermal effect. (**c**) Calcein AM/PI live/dead staining of untreated control cells and MCF-7-ADR cells treated with MoS_2_-PEI + NIR, MoS_2_-PEI-HA + NIR, free DOX, DOX@MoS_2_-PEI-HA, or DOX@MoS_2_-PEI-HA + NIR (equivalent DOX concentration: 25 μM; 808 nm laser, 0.6 W·cm^−2^, 10 min). The predominance of red fluorescence in the DOX@MoS_2_-PEI-HA + NIR group confirms the highest level of cell death and supports the superior efficacy of the combined treatment against drug-resistant cells. Statistical significance: * *p* < 0.05, ** *p* < 0.01. Reprinted with permission from [59]. Copyright 2018 American Chemical Society. Abbreviations: DOX: doxorubicin; HA: hyaluronic acid; MoS_2_: molybdenum disulfide; NIR: near-infrared; PEI: polyethylenimine.

In MCF-7-ADR cells, Biotin-BSA-PEI-LA-MoS_2_-LA-PEG irradiated with an 808 nm laser at 1.5 W·cm^−2^ for 20 min reduced viability to 70% for the material alone and to 15% in the presence of DOX (6 µg·mL^−1^) [58]. Under milder irradiation conditions (808 nm, 0.6 W·cm^−2^, 10 min), MoS_2_-PEI-HA decreased MCF-7-ADR cell viability to 20% at 100 µg·mL^−1^, whereas the DOX-loaded system further reduced viability to 2.9% (Figure 5) [59]. In MCF-7 cells, MoS_2_-RBC irradiated at 808 nm and 2 W·cm^−2^ for 10 min yielded 35% viability at 50 µg·mL^−1^, which decreased to 20% when DOX was incorporated [60]. Similarly, MoS_2_-CS produced marked photothermal cytotoxicity in both KB and Panc-1 cells after 24 h incubation and 7 min of irradiation at 808 nm (1 W·cm^−2^), with viability values of 10% and 25%, respectively, for the material alone, and 5% for the DOX-loaded system in both cell lines [61].

These in vitro chemo-photothermal studies differ in design priority rather than simply in cytotoxic potency. Within the DOX-based group, MoS_2_-PEI-HA appears to advance earlier targeting/stabilisation strategies by achieving very strong activity under comparatively milder irradiation conditions in drug-resistant MCF-7-ADR cells, whereas MoS_2_-RBC prioritises biomimetic stealth behaviour and MoS_2_-CS represents a simpler high-photothermal-output platform evaluated in different tumour cell models. Accordingly, the main distinction among these studies lies in whether the formulation was designed primarily to overcome drug resistance, extend circulation, or maximise local photothermal injury. Because the cell lines, drug concentrations, and irradiation parameters differ across reports, these data should be interpreted as evidence of distinct optimisation routes rather than as a strict efficacy ranking.

Drug- or photosensitiser-assisted effects were also observed for other MoS_2_ formulations. In 4T1 cells, MoS_2_-PEG evaluated under combined photothermal and photodynamic irradiation conditions showed 100% viability for the material alone at 6.4 µg·mL^−1^, whereas the Ce6-containing system reduced viability to 15% after 20 min of treatment [7]. For formulations evaluated without an additional therapeutic cargo, MoS_2_-AuNRs-HAP-PDA and MoS_2_-PEI reduced the viability of MCF-7 and 4T1 cells to 44.85% and 10.94%, respectively, under 808 nm laser irradiation [63,64]. MoS_2_-PEG-Biotin, assessed in A549 cells after 48 h incubation, yielded 41.4% viability for the material alone and 10% when combined with Cur and Er after 10 min of irradiation at 1 W·cm^−2^ [65]. In C6 cells, siRNA/DOX@FA-MoS_2_-peptide reduced viability to approximately 68% for the material alone and to approximately 22% when combined with therapeutic cargo after 48 h incubation and 808 nm irradiation at 1.35 W·cm^−2^ for 3 min [66]. Meanwhile, MoS_2_-AIPH@LAU tested in HT29 cells yielded 32.9% viability for the nanomaterial alone and 6.1% for the AIPH-loaded system after 24 h incubation and 808 nm irradiation at 1 W·cm^−2^ for 5 min [67].

When the broader set of studies is considered, a clear progression in therapeutic logic becomes apparent. Ce6@MoS_2_-PEG couples PTT with PDT, so the added cargo contributes to ROS generation rather than conventional chemotherapy. Cur/Er@MoS_2_-PEG-Biotin shifts toward dual-drug chemo-photothermal co-delivery, while FA-MoS_2_-peptide adds a gene-silencing component and MoS_2_-AIPH@LAU addresses hypoxic tumours through thermally triggered radical generation. By contrast, MoS_2_-AuNRs-HAP-PDA and IR820@MoS_2_-PEI illustrate that some platforms were designed mainly to intensify photothermal or photodynamic output rather than to maximise drug-mediated cytotoxicity.

Across the compiled reports, irradiation was performed predominantly with an 808 nm laser for 5–20 min, while incubation times were usually 24 h. The frequent use of 808 nm likely reflects the historical predominance of NIR-I photothermal protocols, since this wavelength lies within a biologically permissive optical window, is compatible with the strong NIR absorption of many photothermal nanomaterials and can be readily generated with widely used diode-laser systems [78,79]. Exposure times of 5–20 min appear to represent a practical compromise between delivering sufficient thermal dose to induce hyperthermia and limiting excessive overheating or collateral damage. Likewise, the commonly adopted 24 h incubation period probably aims to allow adequate nanoparticle adsorption, cellular internalisation, and early intracellular trafficking before irradiation [80,81].

## 6. MoS_2_/Drug Conjugates for Cancer Phototherapy—In Vivo Studies

In vivo phototherapy studies provide essential insight into the therapeutic potential of MoS_2_-based nanoplatforms under biologically relevant conditions. Unlike in vitro experiments, these studies capture the complex interactions between nanomaterials, tumour tissue, and systemic biological processes, enabling a more comprehensive assessment of treatment efficacy and safety. Therapeutic performance is generally evaluated through tumour growth response following irradiation, complemented by additional indicators such as body-weight variation, tumour-site temperature elevation, and histopathological analysis of tumour tissues. Together, these parameters provide an integrated perspective on the antitumour activity, photothermal performance, and overall tolerability of MoS_2_-based phototherapeutic systems in vivo.

The studies summarised in Table 5 report the in vivo performance of various surface-engineered and drug-conjugated MoS_2_ platforms evaluated in murine tumour models, including MCF-7-ADR, 4T1, Panc-1, and A549 xenografts. Experimental follow-up periods typically ranged from 14 to 25 days, during which tumour growth inhibition and systemic tolerability were monitored. Across these models, many MoS_2_-based formulations demonstrated substantial tumour suppression under near-infrared irradiation, often accompanied by localised temperature increases at the tumour site and histopathological evidence of therapy-induced cellular damage. Importantly, most studies also reported minimal changes in body weight, suggesting that these nanoplatforms can achieve effective phototherapeutic responses while maintaining acceptable systemic safety profiles. Collectively, these findings illustrate the potential of rationally engineered MoS_2_ systems for multimodal cancer phototherapy and highlight the importance of formulation design and irradiation parameters in determining in vivo treatment outcomes.

**Figure 6:**
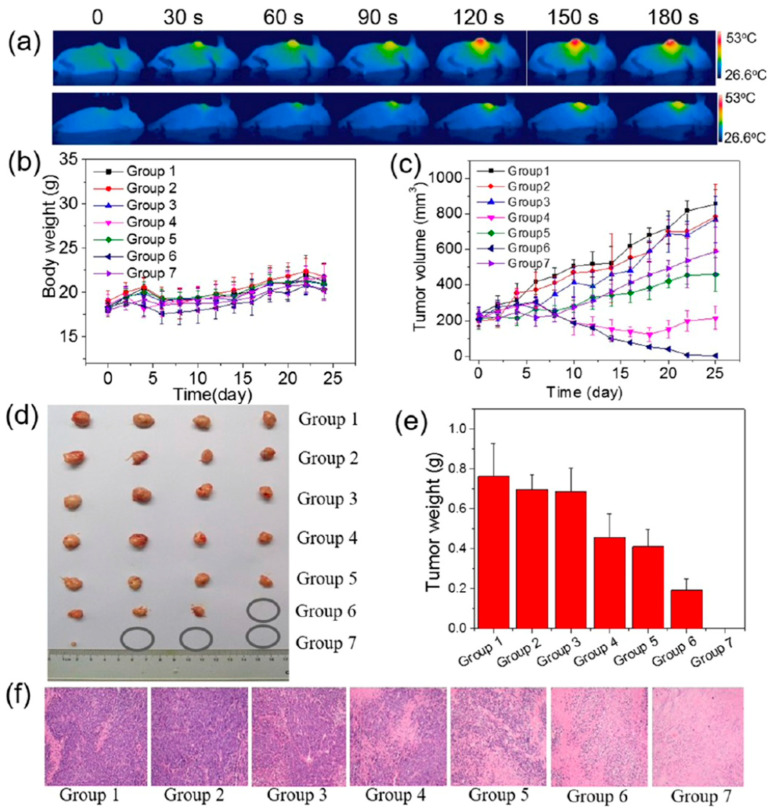
In vivo phototherapy of cancer using MoS_2_-based materials. (**a**) Infrared thermal images of MCF-7-ADR tumour-bearing mice during irradiation with an 808 nm laser (0.6 W·cm^−2^), showing a progressive increase in local temperature to the hyperthermia range, consistent with efficient in vivo photothermal conversion. (**b**) Body weight changes during treatment, showing no substantial weight loss, therefore suggesting low systemic toxicity. (**c**) Tumour volume growth curves. (**d**) Photographs of excised tumours after treatment. (**e**) Average tumour weight. (**f**) H&E-stained tumour sections collected 25 days after injection (group 1: saline; group 2: saline + NIR; group 3: MoS_2_-PEI-HA; group 4: free DOX; group 5: MoS_2_-PEI-HA + NIR; group 6: MoS_2_-PEI-HA/DOX; group 7: MoS_2_-PEI-HA/DOX + NIR). Overall, the DOX-loaded MoS_2_-PEI-HA platform combined with NIR irradiation showed the strongest antitumour effect, as evidenced by the greatest suppression of tumour growth, the smallest excised tumours, the lowest tumour weights, and more pronounced histological tumour damage compared with the control and single-treatment groups. Reprinted with permission from [59]. Copyright 2018 American Chemical Society. Abbreviations: DOX: doxorubicin; H&E: haematoxylin and eosin; HA: hyaluronic acid; MoS_2_: molybdenum disulfide; NIR: near-infrared; PEI: polyethylenimine.

In MCF-7-ADR tumour-bearing BALB/c nude mice, IV administration of DOX@MoS_2_-PEI-HA (10 mg·kg^−1^), followed by 808 nm laser irradiation at 0.6 W·cm^−2^ for 10 min, resulted in 96% tumour growth inhibition, with no tumour regrowth and no significant weight loss. The tumour-site temperature reached approximately 45 °C, and histological examination revealed cell shrinkage and nuclear damage (Figure 6) [59]. In 4T1 tumour-bearing BALB/c mice, IV administration of MoS_2_-RBC loaded with DOX (MoS_2_ 0.8 mg·mL^−1^; DOX 0.75 mg·mL^−1^), followed by 808 nm irradiation at 1.5 W·cm^−2^ for 5 min, produced approximately 96% tumour growth inhibition, again without significant weight loss, while the tumour-site temperature reached 45.3 °C [60]. Similarly, IT administration of MoS_2_-CS loaded with DOX (MoS_2_-CS 2 mg·kg^−1^; DOX 0.95 mg·kg^−1^) in Panc-1 tumour-bearing male BALB/c nude mice, followed by 808 nm laser irradiation at 0.9 W·cm^−2^ for 7 min, yielded approximately 85% tumour growth inhibition, with no significant weight loss. The tumour-site temperature reached approximately 55 °C, and histopathological analysis showed organised vacuolar degeneration, eosinophilic cytoplasm, nuclear damage, and necrosis [61].

Combined photothermal and photodynamic treatment with MoS_2_-PEG (6.85 mg·kg^−1^) and Ce6 (2.0 mg·kg^−1^) in 4T1 tumour-bearing BALB/c mice, using 808 nm irradiation at 0.45 W·cm^−2^ for 20 min (PTT) and 660 nm irradiation at 5 mW·cm^−2^ for 20 min (PDT), resulted in approximately 70% tumour growth inhibition, without significant weight loss. Furthermore, the tumour-site temperature reached 44.8 °C. Meanwhile, PTT and PDT treatments applied individually showed lower efficacy, confirming the power of combination phototherapy [7]. In another 4T1 tumour-bearing BALB/c model, IV administration of IR820@MoS_2_-PEI (100 μg·mL^−1^, 100 µL), followed by 808 nm laser irradiation at 1 W·cm^−2^ for 5 min, achieved 98.3% tumour growth inhibition, with no tumour regrowth and no significant weight loss. The tumour-site temperature reached approximately 56 °C, and severe nuclear damage together with reduced cell proliferation was observed [64]. Likewise, IV administration of MoS_2_-PEG-Biotin (8 mg·kg^−1^) together with curcumin (1.6 mg·kg^−1^) and erlotinib (0.8 mg·kg^−1^) in A549 tumour-bearing BALB/c nude mice, followed by 808 nm laser irradiation at 1 W·cm^−2^ for 10 min, resulted in 95.6% tumour growth inhibition, with no significant weight loss. The tumour-site temperature reached approximately 48 °C, and tumour cell necrosis and lysis were reported [65].

In a C6 syngeneic glioma rat model, IV-administered siRNA/DOX@FA-MoS_2_-peptide followed by 808 nm irradiation at 1 W·cm^−2^ for 10 min produced a 15-fold reduction in tumour volume, with no significant body-weight loss and decreased proliferating cell nuclear antigen (PCNA) expression [66]. In HT29 tumour-bearing BALB/c nude mice, MoS_2_-AIPH@LAU administered by IV or IT routes and irradiated at 808 nm (1 W·cm^−2^, 5 min) achieved complete tumour eradication without recurrence, while body weight remained stable and tumour-site temperature reached approximately 50 °C [67].

These studies are separated into distinct designed categories, as previously mentioned. DOX@MoS_2_-PEI-HA and DOX@MoS_2_-RBC achieved similar tumour growth inhibition, but while the former emphasised targeted, enzyme-/pH-/NIR-responsive release in a drug-resistant breast cancer model, the latter prioritised prolonged circulation and immune evasion. DOX@MoS_2_-CS should again be interpreted differently because IT delivery and higher tumour temperatures favour strong local ablation but provide less information about systemic targeting efficiency. Ce6@MoS_2_-PEG offered proof of concept for combined PTT/PDT, while IR820@MoS_2_-PEI, Cur/Er@MoS_2_-PEG-Biotin, FA-MoS_2_-peptide, and MoS_2_-AIPH@LAU progressively extended the field toward stronger photothermal output, dual-drug delivery, gene-assisted therapy, and oxygen-independent radical therapy. Therefore, the literature reflects diversification of therapeutic strategy more than a simple stepwise increase in efficacy, and direct ranking remains limited by differences in tumour model, administration route, and irradiation conditions.

Across the studies conducted over 14 to 25 days, MoS_2_-based systems, administered mainly intravenously or intratumourally, produced marked antitumour effects after irradiation predominantly at 808 nm for 5 to 20 min at power densities between 0.45 and 1.5 W·cm^−2^. Tumour-site temperatures ranged from 44.8 to approximately 56 °C, with tumour growth inhibition between 70% and 98.3%, and no tumour regrowth was reported in some studies. Treatment was generally well tolerated, as indicated by the absence of significant weight loss. Temperature values are mechanistically relevant because temperatures around 42–45 °C are commonly associated with mild hyperthermia, whereas temperatures above approximately 50 °C are more consistent with direct thermal ablation and necrosis [33,34]. Histopathological findings further supported the therapeutic effect, including cell shrinkage, nuclear damage, necrosis, vacuolar degeneration, eosinophilic cytoplasm, tumour cell lysis, and reduced cell proliferation. Nevertheless, these conclusions should be interpreted as qualitative evidence of platform potential rather than as a quantitative ranking of formulations, because the current literature is dominated by short-term small-animal studies that differ substantially in tumour model, administration route, irradiation conditions, and follow-up duration.

## 7. Challenges and Opportunities

Despite the promising antitumour responses summarised above, the clinical translation of MoS_2_-based phototherapeutic nanoplatforms remains constrained by certain questions regarding their in vivo behaviour. In the studies reviewed here, several IV-administered formulations accumulated in the liver and spleen, indicating that tumour delivery still competes with uptake by the mononuclear phagocyte system [82]. This broader pattern agrees with biodistribution studies showing that PEGylated MoS_2_ nanosheets predominantly accumulate in the liver and spleen after IV injection, although MoS_2_ may subsequently degrade and be excreted more efficiently than some other transition-metal dichalcogenides [83]. In addition, MoS_2_ biodistribution and in vivo behaviour is not determined only by size and surface chemistry, but also by protein-corona formation, which changes with particle size, surface coating and patient-specific variables such as age, sex, ancestry, disease state and comorbidities that can alter uptake, circulation, immune recognition and targeting performance [84]. Apolipoprotein E-rich coronas have been associated with enrichment in liver sinusoid and splenic red pulp, followed by biotransformation of Mo species that can modulate hepatic molybdoenzyme activity, affecting liver metabolism [85]. Generally, nanomedicine studies show that increased particle size, exposed cationic surface groups, and insufficient surface shielding can accelerate sequestration in the liver and spleen [86]. For 2D materials in particular, nano–bio interfacial effects are strongly coupled to lateral dimensions, surface defects, charge density and coating stability [51]. This means that a formulation that appears “stealthy” or targeted under one experimental condition may behave differently in another biological background, and that successful translation will require designs validated across biologically realistic settings rather than idealised cell-culture conditions [87,88]. These insights also underscore the need for tighter control of MoS_2_ dimensions, charge, aggregation state, and coating composition.

Long-term safety is another central challenge. Most in vivo studies reviewed here monitored animals for only up to 25 days and mainly relied on body weight, routine histology, and serum biochemistry. Those endpoints are necessary but insufficient for metal-containing 2D systems, where persistence, dissolution products, repeated-dose exposure, macrophage sequestration, and organ-specific effects can become decisive [89,90]. Importantly, the literature is not fully uniform. Whereas some PEGylated MoS_2_ formulations appear degradable and largely excretable within approximately one month [83], other studies indicate that chronic or mechanistically targeted exposure can reveal liver-specific liabilities. Long-term exposure to MoS_2_ nanosheets has been associated with hepatic lipid accumulation and atherogenesis in apolipoprotein E-deficient mice [91], and more recent mechanistic work showed marked liver accumulation, MST2/Hippo pathway activation, impaired liver regeneration, and autophagy-dependent hepatocyte death [92]. In parallel, studies suggest that MoS_2_ dissolution behaviour and aggregation state strongly influence toxicity, with Kupffer cells showing greater sensitivity than hepatocytes or sinusoidal endothelial cells. Released hexavalent Mo species and aggregated material can drive oxidative stress, apoptosis, lysosomal damage, and inflammasome signalling [93]. Accordingly, future biosafety assessment should extend beyond short-term tolerability and opportunities therefore lie in safety-by-design approaches: ultrasmall or locally retained systems that reduce systemic burden, biodegradable constructs that disassemble under tumour-relevant triggers, and delivery platforms such as hydrogels or microneedles that favour local treatment over prolonged whole-body distribution [67,94,95]. An additional limitation of many intravenously administered MoS_2_ nanoplatforms is their partial reliance on passive tumour accumulation, which is often discussed in the context of the enhanced permeability and retention (EPR) effect. In practice, EPR-mediated delivery is heterogeneous and difficult to predict across tumour types and biological settings, which may limit the translational performance of systemically delivered nanomaterials. In this context, scaffold-based or implantable photothermal systems represent an important complementary strategy because they can localise the photothermal agent directly at the tumour site or within the post-surgical cavity, thereby reducing dependence on systemic biodistribution and passive tumour extravasation. Representative examples include 3D-printed MoS_2_-containing scaffolds developed for simultaneous photothermal tumour ablation and bone regeneration, as well as more recent photothermal composite scaffolds designed to combine local cancer treatment with tissue-engineering functions. Accordingly, scaffold-, hydrogel-, or implant-based local delivery approaches may be particularly attractive for anatomically accessible tumours and postoperative applications, where they may help reduce off-target organ accumulation while improving local therapeutic retention [96,97].

Targeting remains another area where the literature often promises more than biology allows. Ligands, membranes, extracellular vesicles and biomimetic coatings can improve cell affinity and tumour retention, but they do not abolish the sequential barriers of circulation, vascular extravasation, interstitial transport, cellular uptake, endosomal escape and intracellular trafficking [87,94]. This is particularly relevant for multifunctional MoS_2_ systems that carry drugs, nucleic acids or immune modulators, because each added component modifies surface chemistry and manufacturing complexity.

Reproducibility and manufacturing consistency represent an additional barrier to translation. For MoS_2_ systems, critical quality attributes extend well beyond mean size and zeta potential to include thickness, lateral size distribution, crystalline phase, defect density, coating coverage, ligand orientation, drug-loading reproducibility, release kinetics, sterilisation stability and batch-to-batch consistency [54,56]. In nanomedicine, similar batches can differ in size distribution, agglomeration behaviour, impurity profile, coating density, and colloidal stability, and such apparently small variations may materially alter biological responses [98]. This difficulty, along with incomplete reporting across the literature, has prompted calls for minimum information standards such as Minimum Information Reporting in Bio-Nano Experimental Literature (MIRIBEL) and for more standardised analytical characterisation workflows [99,100]. For MoS_2_-based systems, reproducibility is especially important because therapeutic output depends not only on carrier composition but also on drug loading, release kinetics, photothermal conversion efficiency, dispersion stability in physiological media, and irradiation parameters. Quality by design (QbD)-based development, risk assessment, process analytical technologies, model-informed drug development and stronger in vitro–in vivo correlations are crucial to manage these variables before clinical escalation [101,102,103]. Additionally, future studies would benefit from more systematic reporting of exfoliation and synthesis conditions, lateral size and thickness distributions, phase composition, surface chemistry, protein-corona behaviour, batch variability, and light dosimetry.

For MoS_2_ phototherapy, an additional translational issue is that light delivery is part of the product design, not just an afterthought. Dose, wavelength, beam profile, treatment geometry, tissue optical properties and the choice between laser, LED, fibre-coupled, interstitial, wearable or implantable emitters all influence efficacy and safety [10,104]. This matters especially for skin, oral, postoperative and other localised indications, where LED, OLED, hydrogel and microneedle-based platforms may offer a more realistic clinical path than systemic administration followed by deep external irradiation [11,12,13]. The field is therefore moving toward NIR-II-responsive or multimodal systems, local delivery implants, immune-engaging combinations, and device-integrated phototherapy strategies that couple material design to practical dosimetry and patient workflow [105,106].

Regulatory translation poses equally complex challenges. United States Food and Drug Administration (FDA) guidance for drug products containing nanomaterials emphasises that developers should define and control critical nanomaterial quality attributes, including chemical composition, average particle size, particle size distribution, morphology, stability, impurities, and biodegradability, and it recommends characterisation across multiple batches when comparability is being assessed [107]. European Medicines Agency (EMA) guidance similarly highlights lifecycle development issues that require specific consideration for coated nanomedicine products administered parenterally [108]. For MoS_2_-based phototherapy, the regulatory route may be even more complex when the nanoplatform is developed together with a dedicated irradiation system, because the final therapeutic product may raise drug–device combination product questions [107]. Nevertheless, these challenges also define the main opportunities for progress: clearance-by-design formulations, standardised preclinical pipelines, image-guided pharmacokinetic studies, and scalable manufacturing strategies could all make MoS_2_ systems more predictable and regulatorily tractable.

One particularly promising opportunity is to engineer MoS_2_ systems that preserve photothermal or multimodal efficacy while minimising long-term tissue retention. Ultrasmall glutathione-modified MoS_2_ nanodots and, more recently, 2 nm renally excretable MoS_2_ nanoparticles have demonstrated rapid elimination in vivo, illustrating that renal-clearance-oriented design is feasible [109,110]. In parallel, integrating biodistribution, clearance, and degradation studies earlier in formulation development could help identify candidates based not only on in vitro anticancer efficacy, but also on tumour delivery efficiency and whole-body safety. Overall, the next phase of this field will likely depend less on proving that MoS_2_ can participate in formulations that kill tumour cells under irradiation in mice, which is already well established, and more on developing nanoplatforms whose chemistry, biology, device logic, and manufacturing controls survive contact with the real world.

## 8. Conclusions

MoS_2_-based nanomaterials have emerged as highly promising platforms for cancer phototherapy due to the combination of intrinsic photothermal properties, high surface area, chemical versatility, and the capacity to accommodate a wide range of surface modifications and therapeutic cargoes. The studies reviewed here collectively show that rational functionalisation of MoS_2_ greatly influences its behaviour in biological environments, improving parameters such as biocompatibility, cellular internalisation, targeting ability, immune evasion, and controlled drug release. These features make MoS_2_ particularly attractive for the design of multifunctional systems capable of integrating photothermal treatment with chemotherapy or even photodynamic therapy.

The available in vitro and in vivo evidence indicates that most of the reported MoS_2_-based formulations display favourable short-term safety profiles under the tested conditions, while achieving substantial antitumour effects after irradiation. In particular, drug-loaded and surface-engineered systems consistently outperformed non-loaded counterparts, supporting the concept that MoS_2_ is not only an effective PTA but also a versatile carrier for combination therapy. The recurrent observation of tumour growth inhibition together with limited short-term systemic toxicity supports the preclinical therapeutic potential of these nanoplatforms, although these findings should be interpreted as evidence of promising short-term tolerability rather than definitive biosafety. However, additional studies on off-target accumulation, long-term safety, and body clearance are necessary, and direct cross-study comparison remains limited by heterogeneity in tumour models, treatment protocols, and light dosimetry.

At the same time, the literature also suggests that therapeutic performance depends strongly on formulation design, including the nature of the coating, targeting strategy, cargo type, and irradiation conditions. Beyond optimisation of therapeutic efficacy, future progress will also depend on improving batch-to-batch reproducibility, physicochemical standardisation, scalable manufacturing, and light dosimetry, while addressing the regulatory complexity of nanoplatforms developed together with irradiation devices. In this context, strategies based on safety-by-design, clearance-oriented engineering, and local or device-integrated delivery may offer particularly promising routes for translation. Overall, MoS_2_-based drug conjugates represent a valuable and versatile platform for the advancement of cancer phototherapy, with strong potential for the development of more selective and effective therapeutic approaches provided that efficacy is matched by predictable biodistribution, acceptable long-term safety, and robust translational manufacturability.

## Figures and Tables

**Table 1:** Molybdenum disulfide functionalisation and drug conjugation strategies.

Material	Therapeutic Cargo	Functionalisation Strategy	Key Effects	Refs.
Bi otin-BSA-PEI-LA-MoS_2_-LA-PEG	DOX Drug loading: EE = 23% DL = 34% (drug:material ratio = 2.5:1, pH 5.5) Drug release: 15% (pH 7.0, 72 h) 30% (pH 5.5, 72 h) Drug release with irradiation (laser 808 nm, 1.5 W·cm^−2^, 30 min) 23% (pH 7.0, 72 h) 42% (pH 5.5, 72 h)	(1) LA-PEI adsorption on MoS_2_ (2) PEI-LA-MoS_2_-LA-PEG synthesised by adsorption (3) Biotin-BSA obtained by EDC/NHS and adsorption on PEI-LA-MoS_2_-LA-PEG (4) DOX adsorption on Biotin-BSA-PEI-LA-MoS_2_-LA-PEG	Photothermal output unchanged over four laser on/off cycles at 1.5 W·cm^−2^ (10 min heating/10 min cooling per cycle) Increased cellular uptake (biotin-mediated targeting)	[58]
MoS_2_-PEI-HA	DOX Drug loading: DL = 34% (drug:material weight ratio n/s, pH 8.0) Drug release: 11% (pH 7.4, 6 h) 25% (pH 5.0, 6 h) Drug release with HAase: 23% (pH 7.4, 6 h) 42% (pH 5.0, 6 h) Drug release with irradiation (laser 808 nm, 0.6 W·cm^−2^, 10 min) 35.8% (pH 5.0, 6 h) Drug release with irradiation + HAase: 77% (pH 5.0, 6 h)	(1) MoS_2_-PEI formation through electrostatic interaction (2) MoS_2_-PEI-HA synthesised by coupling -NH_2_ with -COOH of HA (3) DOX adsorption on MoS_2_-PEI-HA	Maintained photothermal performance over four 600 s irradiation/cooling cycles at 1.0 W·cm^−2^ Improved biocompatibility (HA) CD44 targeting (via HA) Enzyme-responsive DOX release (HA degradation) Zeta Potential: MoS_2_ = −20 mV MoS_2_-PEI = 20 mV MoS_2_-PEI-HA = −18 mV	[59]
MoS_2_-RBC	DOX Drug loading: DL = 99% (drug:material ratio = 2:1, pH 7.4) Drug release: ~14.5% (pH 5.5, 4 h) ~7% (pH 7.4, 4 h) Drug release with irradiation (laser 808 nm, 2 W·cm^−2^) ~15% (pH 5.5, 4 h) ~8% (pH 7.4, 4 h)	(1) MoS_2_-RBC formation by ultrasonic uniform dispersion (2) DOX adsorption on MoS_2_-RBC	Improved stability in water (evenly dispersed in water, PBS, and RPMI-1640 + 10% serum for 48 h, whereas unmodified MoS_2_ showed visible sedimentation by 24 h) Immune evasion (reduced macrophage phagocytosis and protein adsorption) Zeta Potential: MoS_2_ = −47.97 mV MoS_2_-RBC = −36.43 mV MoS_2_-RBC-DOX = −17.37 mV	[60]
MoS_2_-CS	DOX Drug loading: DL = 32% (drug:material ratio n/s, pH 8.0) Drug release: 6% (pH 8.0, 1 h) Drug release with irradiation (laser 808 nm, 0.8 W·cm^−2^, 10 min) 12.4% (pH 8.0, 1 h)	(1) MoS_2_-CS formation during the exfoliation process (2) DOX is noncovalently loaded onto MoS_2_-CS	High physiological stability (well dispersed in deionized water up to 1 mg·mL^−1^ and stable in various physiological solutions for at least 1 week) Improved biocompatibility Zeta Potential: MoS_2_-CS = 15 mV DOX = −15 mV	[61]
MoS_2_-PDA-poly(MPC-IA)	CDDP Drug loading: DL = 55% (drug:material ratio = 0.27:1, pH 7.4) Drug release: 49% (pH 5.5, 48 h) 15% (pH 7.4, 48 h)	(1) MoS_2_-PDA formation through the self-polymerization of dopamine (2) Surface-initiated SET-LRP of MPC and IA monomers (3) CDDP loaded on via coordination with carboxyl groups	Improved dispersibility in PBS (stable in PBS for at least 24 h; unmodified MoS_2_ precipitated within 5 min, while MoS_2_-PDA was almost completely deposited by 12 h) Zeta Potential: MoS_2_-PDA-poly(MPC-IA) = −32.3 eV	[62]
MoS_2_-PEG	Ce6 Drug loading: DL = 30% (drug:material ratio = 2.5:1, pH 6.0)	(1) PEG adsorption on MoS_2_ (2) MoS_2_-PEG load Ce6 by physical adsorption	Reported as stable in physiological solutions and highly water-soluble after PEGylation Enhanced uptake Generation of ROS	[7]
MoS_2_-AuNRs-HAP-PDA	DOX Drug loading: EE = 80% (drug:material ratio = 0.075:1, pH 7.4) Drug release: 31% (pH 4.5, 10 h) ~12% (pH 7.4, 10 h) Drug release with irradiation (laser 808 nm, 4 W·cm^−2^) 49% (pH 4.5, 10 h) ~15% (pH 7.4, 10 h)	(1) In situ growth of AuNRs on MoS_2_ (2) MoS_2_-AuNRs-HAP-PDA formation by self-assembly of HAP and PDA (3) DOX loaded on MoS_2_-AuNRs-HAP-PDA via PDA adhesion and HAP electrostatic interaction	Increased stability in aqueous solutions (MA remained uniformly dispersed in deionized water for 6 days, whereas MoS_2_ showed distinct aggregation after 3 days) Enhanced PTT efficacy (AuNRs) Zeta Potential: MoS_2_ = −20.63 mV MoS_2_-AuNRs = 44.57 mV MoS_2_-AuNRs-HAP = −2.05 mV MoS_2_-AuNRs-HAP-PDA = −7.45 mV	[63]
MoS_2_-PEI	IR820 Drug loading: DL = 9% (drug:material ratio = 1:3, pH 7.4)	(1) PEI adsorption on MoS_2_ (2) IR820 loading on MoS_2_-PEI via electrostatic interaction	Generation of ROS Zeta Potential: MoS_2_ = −47.6 eV MoS_2_-PEI = 28.3 mV MoS_2_-PEI-IR820 = −19.5 mV	[64]
MoS_2_-PEG-Biotin	Cur Drug loading: DL = 22% (drug:material ratio n/s) Drug release: 7% (2 h) Drug release with irradiation (laser 808 nm, 0.6 W·cm^−2^, 10 min) 17% (2 h) Er Drug loading: DL = 10% (drug:material ratio n/s) Drug release: 5% (2 h) Drug release with irradiation (laser 808 nm, 0.6 W·cm^−2^, 10 min) 12% (2 h)	(1) PEG-Biotin grafted onto MoS_2_ via thiol binding (2) Cur and Er adsorption on MoS_2_-PEG-Biotin	Remarkable physiological stability (well dispersed in DI water, PBS, and cell medium for 7 days) Increased cellular uptake (biotin-mediated targeting) Zeta Potential: MoS_2_ = −44 mV MoS_2_-PEG-Biotin = −36 mV	[65]
FA-MoS_2_-peptide	DOX Drug loading: EE = 30% (drug:material ratio n/s) Drug release: ~63% (8 h) Drug release with irradiation (laser 808 nm, 1.35 W·cm^−2^, 3 min) ~90% (8 h) Anti-Gal-1 siRNA Drug loading: EE = 67% (drug:material ratio n/s)	(1) Bulk MoS_2_ exfoliated in aqueous self-assembled peptide NSs (2) FA conjugated via EDC/NHS (3) DOX post loaded and anti-Gal-1 siRNA loaded electrostatically	Enhanced targeting and cellular uptake (FA) siRNA protection against ribonuclease degradation	[66]
MoS_2_-LAU	AIPH Drug loading: DL = ~70% (drug:material ratio 25:1) Drug release: ~2% (50 min) Drug release with irradiation (laser 808 nm, 1.0 W·cm^−2^, 5 min on and 20 min off cycles) ~17% (50 min)	(1) Hydrothermal MoS_2_ synthesis (2) AIPH physically adsorbed onto MoS_2_ (3) LA coating	Thermo-responsive release (LA) Improved colloidal stability (hydrodynamic diameter showed no significant fluctuation over 5 days in DMEM, PBS, saline, or water) ROS generation in hypoxic environment	[67]

AIPH: 2,2′-Azobis [2-(2-imidazolin-2-yl)propane] dihydrochloride; Gal-1: galectin-1; AuNRs: gold nanorods; BSA: bovine serum albumin; CDDP: cisplatin; Ce6: chlorin e6; CS: chitosan; Cur: curcumin; DL: drug loading capacity; DOX: doxorubicin; EDC: 1-ethyl-3-(3-dimethylaminopropyl)carbodiimide; EE: encapsulation efficiency; Er: erlotinib; FA: folic acid; FITC: fluorescein isothiocyanate; HA: hyaluronic acid; HAase: hyaluronidase; HAP: hydroxyapatite; IA: itaconic acid; IR820: new indocyanine green; LA: lipoic acid; LAU: lauric acid; MoS_2_: molybdenum disulfide; MPC: 2-methacryloyloxyethyl phosphorylcholine; n/s: non-specified; NHS: N-hydroxysuccinimide; PBS: phosphate-buffered saline; PDA: polydopamine; PE: phosphatidylethanolamine; PEG: polyethylene glycol; PEI: polyethyleneimine; PTT: photothermal therapy; RBCs: red blood cells; ROS: reactive oxygen species; SET-LRP: single electron transfer living radical polymerisation; siRNA: small interfering RNA.

**Table 2:** Biocompatibility of MoS_2_-based materials—in vitro studies.

Material	Size	Culture Conditions and Cell Viability	Uptake and Location	Additional Outcomes	Refs.
B iotin-BSA-PEI-LA-MoS_2_-LA-PEG	200 nm (thickness: <10 nm)	HeLa, 24 h incubation ~105% (500 µg·mL^−1^) Concentrations tested: 0–500 µg·mL^−1^	Biotin-mediated endocytosis	-	[58]
MoS_2_-PEI-HA	30–50 nm (thickness: 5–7 nm)	A549, 24 h incubation ~105% (200 µg·mL^−1^) MCF-7, 24 h incubation ~95% (200 µg·mL^−1^) Concentrations tested: 0–200 µg·mL^−1^	CD44-mediated uptake	Negligible haemolysis (800 µg·mL^−1^, 3 h incubation with RBC)	[59]
MoS_2_-RBC	232 nm	MCF-7, 24 h incubation ~101% (50 µg·mL^−1^) Concentrations tested: 2.5–50 µg·mL^−1^	Evenly distributed in cytoplasm	Reduced phagocytosis by macrophages Reduced BSA adsorption	[60]
MoS_2_-CS	80 nm (thickness: 4–6 nm)	KB, 24 h incubation 85% (400 µg·mL^−1^) Panc-1, 24 h incubation ~99% (400 µg·mL^−1^) Concentrations tested: 0–400 µg·mL^−1^	Distributed in cytoplasm	Negligible haemolysis (800 µg·mL^−1^, 3 h incubation with RBC)	[61]
MoS_2_-PDA-poly(MPC-IA)	n/s	L929, 24 h incubation 90% (100 µg·mL^−1^) Concentrations tested: 0–100 µg·mL^−1^	n/s	-	[62]
MoS_2_-PEG	n/s (thickness: 1 nm)	4T1, 12 h incubation 85% (4 mg·mL^−1^) Concentrations tested: 0.25–8 mg·mL^−1^	Distributed in cytoplasm	-	[7]
MoS_2_-AuNRs-HAP-PDA	100–200 nm	EA.hy926, 24 h incubation 85.32% (50 µg·mL^−1^) MCF-7, 24 h incubation 86.92% (50 µg·mL^−1^) Concentrations tested: 0–50 µg·mL^−1^	n/s	-	[63]
MoS_2_-PEI	340 nm	L929, 24 h incubation 91% (125 µg·mL^−1^) 4T1, 24 h incubation 98% (125 µg·mL^−1^) Concentrations tested: 0–125 µg·mL^−1^	Distributed in cytoplasm	-	[64]
MoS_2_-PEG-Biotin	120 nm (thickness: 2.3 nm)	HELF, 72 h incubation 90% (500 µg·mL^−1^) A549, 72 h incubation 90% (500 µg·mL^−1^) Concentrations tested: 25–500 µg·mL^−1^	Biotin-mediated uptake Distributed in cytoplasm	Negligible haemolysis (500 µg·mL^−1^, 2 h incubation with RBC)	[65]
FA-MoS_2_-peptide	n/s	L929, 24 h incubation ~90% (2.5 µg·mL^−1^) Concentrations tested: 0.625–2.5 µg·mL^−1^	FA increased uptake to ~4.2-fold Lysosomal localization initially and substantial escape by 12 h	-	[66]
MoS_2_-LAU	120 nm	L929, 24 h incubation ~95% (200 µg·mL^−1^) Concentrations tested: 0–200 µg·mL^−1^	n/s	Negligible haemolysis (200 µg·mL^−1^, 2 h incubation with RBC)	[67]

4T1: murine breast adenocarcinoma cell line; A549: adenocarcinomic human alveolar basal epithelial cell line; AuNRs: gold nanorods; BSA: bovine serum albumin; CD44: HA-binding cell adhesion receptor; CS: chitosan; EA.hy926: immortalised human vascular endothelial cell line; FA: folic acid; FITC: fluorescein isothiocyanate; HA: hyaluronic acid; HAP: hydroxyapatite; HeLa: immortalised human cervical epithelial carcinoma cell line; HELF: human embryonic lung fibroblast cell line; IA: itaconic acid; KB: HeLa-derived epithelial carcinoma cell line; L929: murine fibroblast cell line; LA: lipoic acid; LAU: lauric acid; MCF-7: human breast adenocarcinoma cell line; MoS_2_: molybdenum disulfide; MPC: 2-methacryloyloxyethyl phosphorylcholine; n/s: non-specified; Panc-1: human pancreatic ductal adenocarcinoma cell line; PDA: polydopamine; PE: phosphatidylethanolamine; PEG: polyethylene glycol; PEI: polyethyleneimine; RBCs: red blood cells.

**Table 3:** Biocompatibility of MoS_2_-based materials—in vivo studies.

Material	Animal Model	Toxicokinetics	Histology	Additional Observations	Refs.
Mo S_2_-PEI-HA	Female BALB/c mice (15 days)	IV administration (10 mg·kg^−1^) Accumulation in liver, spleen, lung, and tumour	No damage or inflammation in major organs	No significant weight loss	[59]
MoS_2_-RBC	BALB/c mice (24 h)	IV administration (0.8 mg·mL^−1^, 200 μL) Long circulation Accumulation in liver, spleen, and tumour	-	No significant weight loss	[60]
MoS_2_-CS	Male BALB/c nude mice (24 days)	IT administration (2 mg·kg^−1^)	-	No significant weight loss	[61]
MoS_2_-PEG	4T1 tumour-bearing BALB/c mice (21 days)	IV administration (6.85 mg·kg^−1^) Accumulation in tumour	No damage or inflammation in major organs	No significant weight loss	[7]
MoS_2_-PEI	4T1 tumour-bearing BALB/c mice (14 days)	IV administration (100 μg·mL^−1^, 100 µL) Accumulation in tumour Normal serum biochemistry markers	No damage or inflammation in major organs	No significant weight loss	[64]
MoS_2_-PEG-Biotin	A549 tumour-bearing BALB/c nude female mice (21 days)	IV administration (8 mg·kg^−1^) Accumulation in liver, spleen, and tumour	No damage or inflammation in major organs	No significant weight loss	[65]
FA-MoS_2_-peptide	Male Wistar rats (7 days)	IV administration Accumulation in liver, kidney, lung, spleen, brain, and tumour	No damage or inflammation in major organs	No significant weight loss	[66]
MoS_2_-LAU	Healthy KM mice (14 days)	IV administration (1 mg·mL^−1^, 200 µL) Accumulation in liver, spleen, and tumour	No damage or inflammation in major organs	No significant weight loss	[67]

4T1: murine breast adenocarcinoma cell line; A549: adenocarcinomic human alveolar basal epithelial cell line; BALB/c: inbred mouse strain; CS: chitosan; FA: folic acid; HA: hyaluronic acid; IT: intratumoural; IV: intravenous; ID: injected dose; KM: Kunming; LA: lauric acid; LAU: lauric acid; n/s: non-specified; MoS_2_: molybdenum disulfide; PEG: polyethylene glycol; PEI: polyethyleneimine; RBCs: red blood cells.

**Table 4:** MoS_2_/drug conjugates for cancer phototherapy—in vitro studies.

Material	Irradiation Method	Energy	Irradiation Time	Culture conditions and Cell Viability	Refs.
DO X@Biotin-BSA-PEI-LA-MoS_2_-LA-PEG	Laser (808 nm)	1.5 W·cm^−2^	20 min	MCF-7-ADR, 24 h incubation 2DnMat only: 70% 2DnMat + drug: 15% (DOX 6 µg·mL^−1^) Concentrations tested (DOX): 0.1–6 µg·mL^−1^	[58]
DOX@MoS_2_-PEI-HA	Laser (808 nm)	0.6 W·cm^−2^	10 min	MCF-7-ADR, 24 h incubation 2DnMat only: 20% (100 µg·mL^−1^) 2DnMat + drug: 2.9% (DOX 25 µM) Concentrations tested: 25–100 µg·mL^−1^	[59]
DOX@MoS_2_-RBC	Laser (808 nm)	2 W·cm^−2^	10 min	MCF-7, 24 h incubation 2DnMat only: 35% (50 µg·mL^−1^) 2DnMat + drug: 20% (DOX 25 µg·mL^−1^) Concentrations tested: 12.5–50 µg·mL^−1^	[60]
DOX@MoS_2_-CS	Laser (808 nm)	1 W·cm^−2^	7 min	KB, 24 h incubation 2DnMat only: 10% (50 µg·mL^−1^) 2DnMat + drug: 5% (DOX 50 µg·mL^−1^) Panc-1, 24 h incubation 2DnMat only: 25% (50 µg·mL^−1^) 2DnMat + drug: 5% (DOX 50 µg·mL^−1^) Concentrations tested: 0–50 µg·mL^−1^	[61]
Ce6@MoS_2_-PEG	PTT—Laser (808 nm) PDT—Light (660 nm)	0.45 W·cm^−2^ (PTT) 0.005 W·cm^−2^ (PDT)	20 min	4T1, 24 h incubation 2DnMat only: 100% (6.4 µg·mL^−1^) 2DnMat + drug: 15% (Ce6 2 µM) Concentrations tested: 0–50 µg·mL^−1^	[7]
DOX@MoS_2_-AuNRs-HAP-PDA	Laser (808 nm)	0.8 W·cm^−2^	n/s	MCF-7, 24 h incubation 44.85% (50 µg·mL^−1^) Concentrations tested: 0–50 µg·mL^−1^	[63]
IR820@MoS_2_-PEI	Laser (808 nm)	1 W·cm^−2^	5 min	4T1, 24 h incubation 10.94% (125 µg·mL^−1^) Concentrations tested: 0–125 µg·mL^−1^	[64]
Cur/Er@MoS_2_-PEG-Biotin	Laser (808 nm)	1 W·cm^−2^	10 min	A549, 48 h incubation 2DnMat only: 41.4% (100 µg·mL^−1^) 2DnMat + drug: 10% (Cur 20 µg·mL^−1^, Er 10 µg·mL^−1^)	[65]
siRNA/DOX@FA-MoS_2_-peptide	Laser (808 nm)	1.35 W·cm^−2^	3 min	C6, 48 h incubation 2DnMat only: ~68% (2.5 µg·mL^−1^) 2DnMat + drug: ~22% (2.5 µg·mL^−1^)	[66]
MoS_2_-AIPH@LAU	Laser (808 nm)	1 W·cm^−2^	5 min	HT29, 24 h incubation 2DnMat only: 32.9% (200 µg·mL^−1^) 2DnMat + drug: 6.1% (200 µg·mL^−1^)	[67]

2DnMat: 2D-nanomaterial; 4T1: murine breast adenocarcinoma cell line; A549: adenocarcinomic human alveolar basal epithelial cell line; AIPH: 2,2′-Azobis [2-(2-imidazolin-2-yl)propane] dihydrochloride; AuNRs: gold nanorods; BSA: bovine serum albumin; C6: rat-derived glioma cell line; Ce6: chlorin e6; CS: chitosan; Cur: curcumin; DOX: doxorubicin; Er: erlotinib; FA: folic acid; HA: hyaluronic acid; HAP: hydroxyapatite; HT29: human colon adenocarcinoma cell line; KB: HeLa-derived epithelial carcinoma cell line; LA: lipoic acid; LAU: lauric acid; MCF-7: human breast adenocarcinoma cell line; MCF-7-ADR: adriamycin-resistant human breast adenocarcinoma cell line; MoS_2_: molybdenum disulfide; n/s: non-specified; Panc-1: human pancreatic ductal adenocarcinoma cell line; PDA: polydopamine; PDT: photodynamic therapy; PEG: polyethylene glycol; PEI: polyethyleneimine; PTT: photothermal therapy; RBCs: red blood cells; siRNA: small interfering RNA.

**Table 5:** MoS_2_/drug conjugates for cancer phototherapy—in vivo studies.

Material	Irradiation Conditions	Animal Model	Tumour Growth	Additional Observations	Refs.
D OX@MoS_2_-PEI-HA	Laser (808 nm) 0.6 W·cm^−2^ 10 min	MCF-7-ADR tumour-bearing BALB/c nude mice (25 days)	IV administration (10 mg·kg^−1^) Tumour growth inhibition: 96% No tumour regrowth	No significant weight loss Tumour-site temperature reached ~45 °C Cell shrinkage and nuclear damage	[59]
DOX@MoS_2_-RBC	Laser (808 nm) 1.5 W·cm^−2^ 5 min	4T1 tumour-bearing BALB/c mice (15 days)	IV administration (MoS_2_ 0.8 mg·mL^−1^, DOX 0.75 mg·mL^−1^) Tumour growth inhibition: ~96%	No significant weight loss Tumour-site temperature reached 45.3 °C	[60]
DOX@MoS_2_-CS	Laser (808 nm) 0.9 W·cm^−2^ 7 min	Panc-1 tumour-bearing male BALB/c nude mice (24 days)	IT administration (MoS_2_-CS 2 mg·kg^−1^, DOX 0.95 mg·kg^−1^) Tumour growth inhibition: ~85%	No significant weight loss Tumour-site temperature reached ~55 °C Organised vacuolar degeneration, eosinophilic cytoplasm, nuclear damage, and necrosis	[61]
Ce6@MoS_2_-PEG	PTT—Laser (808 nm) 0.45 W·cm^−2^ 20 min PDT—Light (660 nm) 0.005 W·cm^−2^ 20 min	4T1 tumour-bearing BALB/c mice (21 days)	IV administration (6.85 mg·kg^−1^, Ce6 2.0 mg·kg^−1^) Tumour growth inhibition: ~70%	No significant weight loss Tumour-site temperature reached 44.8 °C	[7]
IR820@MoS_2_-PEI	Laser (808 nm) 1 W·cm^−2^ 5 min	4T1 tumour-bearing BALB/c mice (14 days)	IV administration (100 μg·mL^−1^, 100 µL) Tumour growth inhibition: 98.3% No tumour regrowth	No significant weight loss Tumour-site temperature reached ~56 °C Severe nuclear damage and reduced cell proliferation	[64]
Cur/Er@MoS_2_-PEG-Biotin	Laser (808 nm) 1 W·cm^−2^ 10 min	A549 tumour-bearing BALB/c nude mice (21 days)	IV administration (MoS_2_-PEG-Biotin 8 mg·kg^−1^, Cur 1.6 mg·kg^−1^, Er 0.8 mg·kg^−1^) Tumour growth inhibition: 95.6%	No significant weight loss Tumour-site temperature reached ~48 °C Tumour cell necrosis and lysis	[65]
siRNA/DOX@FA-MoS_2_-peptide	Laser (808 nm) 1 W·cm^−2^ 10 min	C6 syngeneic glioma rats (21 days)	IV administration 15-fold reduction in tumour volume	No significant weight loss Decreased expression of PCNA	[66]
MoS_2_-AIPH@LAU	Laser (808 nm) 1 W·cm^−2^ 5 min	HT29 tumour-bearing BALB/c nude mice (14 days)	IV/IT administration Complete tumour eradication No recurrence	No significant weight loss Tumour-site temperature reached ~50 °C	[67]

4T1: murine breast adenocarcinoma cell line; A549: adenocarcinomic human alveolar basal epithelial cell line; AIPH: 2,2′-Azobis [2-(2-imidazolin-2-yl)propane] dihydrochloride; BALB/c: inbred mouse strain; C6: rat-derived glioma cell line; Ce6: chlorin e6; CS: chitosan; Cur: curcumin; DOX: doxorubicin; Er: erlotinib; FA: folic acid; HT29: human colon adenocarcinoma cell line; HA: hyaluronic acid; IT: intratumoural; IV: intravenous; IA: itaconic acid; LA: lipoic acid; LAU: lauric acid; MCF-7-ADR: adriamycin-resistant human breast adenocarcinoma cell line; MoS_2_: molybdenum disulfide; Panc-1: human pancreatic ductal adenocarcinoma cell line; PCNA: proliferating cell nuclear antigen; PDT: photodynamic therapy; PEG: polyethylene glycol; PEI: polyethyleneimine; PTT: photothermal therapy; RBC: red blood cell; siRNA: small interfering RNA.

## Data Availability

Data sharing is not applicable. No new data were created or analyzed in this study.

## References

[1] Brown J.S., Amend S.R., Austin R.H., Gatenby R.A., Hammarlund E.U., Pienta K.J. (2023). Updating the Definition of Cancer. Mol. Cancer Res..

[2] Saini A., Kumar M., Bhatt S., Saini V., Malik A. (2020). Cancer causes and treatments. Int. J. Pharm. Sci. Res.

[3] Bray F., Laversanne M., Sung H., Ferlay J., Siegel R.L., Soerjomataram I., Jemal A. (2024). Global cancer statistics 2022: GLOBOCAN estimates of incidence and mortality worldwide for 36 cancers in 185 countries. CA Cancer J. Clin..

[4] Zafar A., Khatoon S., Khan M.J., Abu J., Naeem A. (2025). Advancements and limitations in traditional anti-cancer therapies: A comprehensive review of surgery, chemotherapy, radiation therapy, and hormonal therapy. Discov. Oncol..

[5] Cai Y., Chai T., Nguyen W., Liu J., Xiao E., Ran X., Ran Y., Du D., Chen W., Chen X. (2025). Phototherapy in cancer treatment: Strategies and challenges. Signal Transduct. Target. Ther..

[6] Montaseri H., Kruger C.A., Abrahamse H. (2021). Targeted photodynamic therapy using alloyed nanoparticle-conjugated 5-aminolevulinic acid for breast cancer. Pharmaceutics.

[7] Liu T., Wang C., Cui W., Gong H., Liang C., Shi X., Li Z., Sun B., Liu Z. (2014). Combined photothermal and photodynamic therapy delivered by PEGylated MoS_2_ nanosheets. Nanoscale.

[8] Ming L., Cheng K., Chen Y., Yang R., Chen D. (2021). Enhancement of tumor lethality of ROS in photodynamic therapy. Cancer Med..

[9] Wang J., Wu X., Shen P., Wang J., Shen Y., Shen Y., Webster T.J., Deng J. (2020). Applications of inorganic nanomaterials in photothermal therapy based on combinational cancer treatment. Int. J. Nanomed..

[10] Algorri J.F., López-Higuera J.M., Rodríguez-Cobo L., Cobo A. (2023). Advanced Light Source Technologies for Photodynamic Therapy of Skin Cancer Lesions. Pharmaceutics.

[11] Mallidi S., Mai Z., Rizvi I., Hempstead J., Arnason S., Celli J., Hasan T. (2015). In vivo evaluation of battery-operated light-emitting diode-based photodynamic therapy efficacy using tumor volume and biomarker expression as endpoints. J. Biomed. Opt..

[12] Liu H., Daly L., Rudd G., Khan A.P., Mallidi S., Liu Y., Cuckov F., Hasan T., Celli J.P. (2019). Development and evaluation of a low-cost, portable, LED-based device for PDT treatment of early-stage oral cancer in resource-limited settings. Lasers Surg. Med..

[13] Tiwari R.K., Mishra R., Sharma S.K., Prabhu N., Nagar M.R., Grigalevicius S. (2025). Advancing Cancer Treatment and Diagnosis: A Review on Photodynamic Therapy Using OLED Technology. Molecules.

[14] Banerjee S.M., El-Sheikh S., Malhotra A., Mosse C.A., Parker S., Williams N.R., MacRobert A.J., Hamoudi R., Bown S.G., Keshtgar M.R. (2020). Photodynamic therapy in primary breast cancer. J. Clin. Med..

[15] Algorri J.F., Ochoa M., Roldan-Varona P., Rodriguez-Cobo L., López-Higuera J.M. (2021). Light technology for efficient and effective photodynamic therapy: A critical review. Cancers.

[16] Kwiatkowski S., Knap B., Przystupski D., Saczko J., Kędzierska E., Knap-Czop K., Kotlińska J., Michel O., Kotowski K., Kulbacka J. (2018). Photodynamic therapy–mechanisms, photosensitizers and combinations. Biomed. Pharmacother..

[17] Dudzik T., Domański I., Makuch S. (2024). The impact of photodynamic therapy on immune system in cancer—An update. Front. Immunol..

[18] Han Y., Tian X., Zhai J., Zhang Z. (2024). Clinical application of immunogenic cell death inducers in cancer immunotherapy: Turning cold tumors hot. Front. Cell Dev. Biol..

[19] Lin L., Song X., Dong X., Li B. (2021). Nano-photosensitizers for enhanced photodynamic therapy. Photodiagn. Photodyn. Ther..

[20] Aebisher D., Szpara J., Bartusik-Aebisher D. (2024). Advances in Medicine: Photodynamic Therapy. Int. J. Mol. Sci..

[21] Merlin J.P.J., Crous A., Abrahamse H. (2024). Combining Photodynamic Therapy and Targeted Drug Delivery Systems: Enhancing Mitochondrial Toxicity for Improved Cancer Outcomes. Int. J. Mol. Sci..

[22] da Silva D.B., da Silva C.L., Davanzo N.N., da Silva Souza R., Correa R.J., Tedesco A.C., Pierre M.B.R. (2021). Protoporphyrin IX (PpIX) loaded PLGA nanoparticles for topical Photodynamic Therapy of melanoma cells. Photodiagn. Photodyn. Ther..

[23] Deng X., Shao Z., Zhao Y. (2021). Solutions to the Drawbacks of Photothermal and Photodynamic Cancer Therapy. Adv. Sci..

[24] Huis In’t Veld R.V., Heuts J., Ma S., Cruz L.J., Ossendorp F.A., Jager M.J. (2023). Current Challenges and Opportunities of Photodynamic Therapy against Cancer. Pharmaceutics.

[25] Xiong T., Chen Y., Li M., Chen X., Peng X. (2025). Recent Progress of Molecular Design in Organic Type I Photosensitizers. Small.

[26] Zuo T., Li X., Ma X., Zhang Y., Li X., Fan X., Gao M., Xia D., Cheng H. (2024). Engineering tumor-oxygenated nanomaterials: Advancing photodynamic therapy for cancer treatment. Front. Bioeng. Biotechnol..

[27] Liu X., Lu Y., Li X., Luo L., You J. (2024). Nanoplatform-enhanced photodynamic therapy for the induction of immunogenic cell death. J. Control. Release.

[28] Thiruppathi J., Vijayan V., Park I.K., Lee S.E., Rhee J.H. (2024). Enhancing cancer immunotherapy with photodynamic therapy and nanoparticle: Making tumor microenvironment hotter to make immunotherapeutic work better. Front. Immunol..

[29] Liu M., Zhu H., Wang Y., Sevencan C., Li B.L. (2021). Functionalized MoS_2_-based nanomaterials for cancer phototherapy and other biomedical applications. ACS Mater. Lett..

[30] Wang J., Sui L., Huang J., Miao L., Nie Y., Wang K., Yang Z., Huang Q., Gong X., Nan Y. (2021). MoS_2_-based nanocomposites for cancer diagnosis and therapy. Bioact. Mater..

[31] Jiang W., Lin L., Wu P., Lin H., Sui J. (2024). Near-Infrared-II Nanomaterials for Activatable Photodiagnosis and Phototherapy. Chem.–Eur. J..

[32] Li H., Li P., Zhang J., Lin Z., Bai L., Shen H. (2024). Applications of nanotheranostics in the second near-infrared window in bioimaging and cancer treatment. Nanoscale.

[33] Yang G.-G., Zhou D.-J., Pan Z.-Y., Yang J., Zhang D.-Y., Cao Q., Ji L.-N., Mao Z.-W. (2019). Multifunctional low-temperature photothermal nanodrug with in vivo clearance, ROS-Scavenging and anti-inflammatory abilities. Biomaterials.

[34] Wang P., Chen B., Zhan Y., Wang L., Luo J., Xu J., Zhan L., Li Z., Liu Y., Wei J. (2022). Enhancing the efficiency of mild-temperature photothermal therapy for cancer assisting with various strategies. Pharmaceutics.

[35] Cai Y., Lv Z., Chen X., Jin K., Mou X. (2024). Recent advances in biomaterials based near-infrared mild photothermal therapy for biomedical application: A review. Int. J. Biol. Macromol..

[36] Lukácsi S., Munkácsy G., Győrffy B. (2024). Harnessing Hyperthermia: Molecular, Cellular, and Immunological Insights for Enhanced Anticancer Therapies. Integr. Cancer Ther..

[37] Premji T.P., Dash B.S., Das S., Chen J.P. (2024). Functionalized Nanomaterials for Inhibiting ATP-Dependent Heat Shock Proteins in Cancer Photothermal/Photodynamic Therapy and Combination Therapy. Nanomaterials.

[38] Debnath M., Debnath S.K., Talpade M.V., Bhatt S., Gupta P.P., Srivastava R. (2024). Surface engineered nanohybrids in plasmonic photothermal therapy for cancer: Regulatory and translational challenges. Nanotheranostics.

[39] Costa-Almeida R., Bogas D., Fernandes J.R., Timochenco L., Silva F.A., Meneses J., Gonçalves I.C., Magalhães F.D., Pinto A.M. (2020). Near-infrared radiation-based mild photohyperthermia therapy of non-melanoma skin cancer with PEGylated reduced nanographene oxide. Polymers.

[40] Cheng Z., Li M., Dey R., Chen Y. (2021). Nanomaterials for cancer therapy: Current progress and perspectives. J. Hematol. Oncol..

[41] Zhang R., Yan Z., Gao M., Zheng B., Yue B., Qiu M. (2024). Recent advances in two-dimensional materials for drug delivery. J. Mater. Chem. B.

[42] Kuang F., Hui T., Chen Y., Qiu M., Gao X. (2024). Post-Graphene 2D Materials: Structures, Properties, and Cancer Therapy Applications. Adv. Healthc. Mater..

[43] Li C., Fang X., Zhang H., Zhang B. (2024). Recent Advances of Emerging Metal-Containing Two-Dimensional Nanomaterials in Tumor Theranostics. Int. J. Nanomed..

[44] Mohammadpour Z., Majidzadeh-A K. (2020). Applications of two-dimensional nanomaterials in breast cancer theranostics. ACS Biomater. Sci. Eng..

[45] Silva F., Chang H.P., Incorvia J.A.C., Oliveira M.J., Sarmento B., Santos S.G., Magalhães F.D., Pinto A.M. (2024). 2D Nanomaterials and Their Drug Conjugates for Phototherapy and Magnetic Hyperthermia Therapy of Cancer and Infections. Small.

[46] Luo Y., Chen M., Zhang T., Peng Q. (2024). 2D nanomaterials-based delivery systems and their potentials in anticancer synergistic photo-immunotherapy. Colloids Surf. B Biointerfaces.

[47] Parihar A., Gaur K., Sarbadhikary P. (2025). Advanced 2D Nanomaterials for Phototheranostics of Breast Cancer: A Paradigm Shift. Adv. Biol..

[48] Rafieerad A., Saleth L.R., Khanahmadi S., Amiri A., Alagarsamy K.N., Dhingra S. (2025). Periodic Table of Immunomodulatory Elements and Derived Two-Dimensional Biomaterials. Adv. Sci..

[49] Sukur S., Ranc V. (2025). Magnetic 2D Transition-Metal-Based Nanomaterials in Biomedicine: Opportunities and Challenges in Cancer Therapy. Materials.

[50] Cheng L., Wang X., Gong F., Liu T., Liu Z. (2020). 2D nanomaterials for cancer theranostic applications. Adv. Mater..

[51] Wang Y., Zhang X., Yue H. (2024). Two-dimensional nanomaterials induced nano-bio interfacial effects and biomedical applications in cancer treatment. J. Nanobiotechnol..

[52] Pourmadadi M., Tajiki A., Hosseini S.M., Samadi A., Abdouss M., Daneshnia S., Yazdian F. (2022). A comprehensive review of synthesis, structure, properties, and functionalization of MoS_2_; emphasis on drug delivery, photothermal therapy, and tissue engineering applications. J. Drug Deliv. Sci. Technol..

[53] Chakraborty G., Padmashree R., Prasad A. (2023). Recent advancement of surface modification techniques of 2-D nanomaterials. Mater. Sci. Eng. B.

[54] Yu X., Xu C., Sun J., Xu H., Huang H., Gan Z., George A., Ouyang S., Liu F. (2024). Recent developments in two-dimensional molybdenum disulfide-based multimodal cancer theranostics. J. Nanobiotechnol..

[55] Ghosh S., Lai J.Y. (2024). An insight into the dual role of MoS2-based nanocarriers in anticancer drug delivery and therapy. Acta Biomater..

[56] Sasanipoor F., Zhang Z. (2025). Molybdenum Disulfide Nanocomposites for Cancer Diagnosis and Therapeutics: Biosensors, Bioimaging, and Phototherapy. Adv. Healthc. Mater..

[57] Samy O., Zeng S., Birowosuto M.D., El Moutaouakil A. (2021). A Review on MoS2 Properties, Synthesis, Sensing Applications and Challenges. Crystals.

[58] Liu J., Lu K., Gao F., Zhao L., Li H., Jiang Y. (2022). Multifunctional MoS_2_ composite nanomaterials for drug delivery and synergistic photothermal therapy in cancer treatment. Ceram. Int..

[59] Dong X., Yin W., Zhang X., Zhu S., He X., Yu J., Xie J., Guo Z., Yan L., Liu X. (2018). Intelligent MoS_2_ nanotheranostic for targeted and enzyme-/pH-/NIR-responsive drug delivery to overcome cancer chemotherapy resistance guided by PET imaging. ACS Appl. Mater. Interfaces.

[60] Li J.-Q., Zhao R.-X., Yang F.-M., Qi X.-T., Ye P.-K., Xie M. (2022). An erythrocyte membrane-camouflaged biomimetic nanoplatform for enhanced chemo-photothermal therapy of breast cancer. J. Mater. Chem. B.

[61] Yin W., Yan L., Yu J., Tian G., Zhou L., Zheng X., Zhang X., Yong Y., Li J., Gu Z. (2014). High-throughput synthesis of single-layer MoS_2_ nanosheets as a near-infrared photothermal-triggered drug delivery for effective cancer therapy. ACS Nano.

[62] Zeng G., Chen T., Huang L., Liu M., Jiang R., Wan Q., Dai Y., Wen Y., Zhang X., Wei Y. (2018). Surface modification and drug delivery applications of MoS_2_ nanosheets with polymers through the combination of mussel inspired chemistry and SET-LRP. J. Taiwan Inst. Chem. Eng..

[63] Wang W., Wang J., Li J., Cao S., Shi J. (2023). In situ growth of MoS_2_@ AuNRs nanoparticles with synergistically enhanced NIR response for controlled drug release. Mater. Today Commun..

[64] Liu J., Cui E., Zhang Q., Xie D. (2024). MoS_2_-based nanocomposites with high photothermal conversion efficiency for combinational photothermal/photodynamic tumor therapy. J. Alloys Compd..

[65] Chen Z., Wei X., Zheng Y., Zhang Z., Gu W., Liao W., Zhang H., Wang X., Liu J., Li H. (2023). Targeted co-delivery of curcumin and erlotinib by MoS_2_ nanosheets for the combination of synergetic chemotherapy and photothermal therapy of lung cancer. J. Nanobiotechnol..

[66] Chibh S., Aggarwal N., Gupta N., Ali S., Mishra J., Tiwari S., Ali M.E., Mishra D.P., Panda J.J. (2025). Photoresponsive and Shape-Switchable MoS_2_–Peptide-Hybrid Nanosystems for Enacting Photochemo and siRNA-Mediated Gene Therapy in Glioma. ACS Appl. Mater. Interfaces.

[67] Yin Y., Wang N., Hu B., Guo J., Chen Q., Chen Z., Shahbazi M.-A., Agüero L., Wang S., Li C. (2025). Thermo-responsive and biodegradable MoS_2_-based nanoplatform for tumor therapy and postoperative wound management. J. Colloid Interface Sci..

[68] Gonçalves M., Mignani S., Rodrigues J., Tomás H. (2020). A glance over doxorubicin based-nanotherapeutics: From proof-of-concept studies to solutions in the market. J. Control. Release.

[69] Murali A., Lokhande G., Deo K.A., Brokesh A., Gaharwar A.K. (2021). Emerging 2D nanomaterials for biomedical applications. Mater. Today.

[70] Kyriakides T.R., Raj A., Tseng T.H., Xiao H., Nguyen R., Mohammed F.S., Halder S., Xu M., Wu M.J., Bao S. (2021). Biocompatibility of nanomaterials and their immunological properties. Biomed. Mater..

[71] Gruber S., Nickel A. (2023). Toxic or not toxic? The specifications of the standard ISO 10993-5 are not explicit enough to yield comparable results in the cytotoxicity assessment of an identical medical device. Front. Med. Technol..

[72] Van Haute D., Liu A.T., Berlin J.M. (2018). Coating Metal Nanoparticle Surfaces with Small Organic Molecules Can Reduce Nonspecific Cell Uptake. ACS Nano.

[73] Qie Y., Yuan H., von Roemeling C.A., Chen Y., Liu X., Shih K.D., Knight J.A., Tun H.W., Wharen R.E., Jiang W. (2016). Surface modification of nanoparticles enables selective evasion of phagocytic clearance by distinct macrophage phenotypes. Sci. Rep..

[74] Zheng H., Mortensen L.J., Ravichandran S., Bentley K., DeLouise L.A. (2017). Effect of Nanoparticle Surface Coating on Cell Toxicity and Mitochondria Uptake. J. Biomed. Nanotechnol..

[75] Almalik A., Benabdelkamel H., Masood A., Alanazi I.O., Alradwan I., Majrashi M.A., Alfadda A.A., Alghamdi W.M., Alrabiah H., Tirelli N. (2017). Hyaluronic Acid Coated Chitosan Nanoparticles Reduced the Immunogenicity of the Formed Protein Corona. Sci. Rep..

[76] Almalik A., Alradwan I., Majrashi M.A., Alsaffar B.A., Algarni A.T., Alsuabeyl M.S., Alrabiah H., Tirelli N., Alhasan A.H. (2018). Cellular responses of hyaluronic acid-coated chitosan nanoparticles. Toxicol. Res..

[77] Wu J., Yu Y., Su G. (2022). Safety assessment of 2D MXenes: In vitro and in vivo. Nanomaterials.

[78] Zhang Y., Zhang S., Zhang Z., Ji L., Zhang J., Wang Q., Guo T., Ni S., Cai R., Mu X. (2021). Recent Progress on NIR-II Photothermal Therapy. Front. Chem..

[79] Wu X., Suo Y., Shi H., Liu R., Wu F., Wang T., Ma L., Liu H., Cheng Z. (2020). Deep-Tissue Photothermal Therapy Using Laser Illumination at NIR-IIa Window. Nanomicro Lett..

[80] Toscano F., Torres-Arias M. (2023). Nanoparticles cellular uptake, trafficking, activation, toxicity and in vitro evaluation. Curr. Res. Immunol..

[81] Donahue N.D., Acar H., Wilhelm S. (2019). Concepts of nanoparticle cellular uptake, intracellular trafficking, and kinetics in nanomedicine. Adv. Drug Deliv. Rev..

[82] Ngo W., Ahmed S., Blackadar C., Bussin B., Ji Q., Mladjenovic S.M., Sepahi Z., Chan W.C.W. (2022). Why nanoparticles prefer liver macrophage cell uptake in vivo. Adv. Drug Deliv. Rev..

[83] Hao J., Song G., Liu T., Yi X., Yang K., Cheng L., Liu Z. (2017). In Vivo Long-Term Biodistribution, Excretion, and Toxicology of PEGylated Transition-Metal Dichalcogenides MS_2_ (M = Mo, W, Ti) Nanosheets. Adv. Sci..

[84] Cisneros E.P., Morse B.A., Savk A., Malik K., Peppas N.A., Lanier O.L. (2024). The role of patient-specific variables in protein corona formation and therapeutic efficacy in nanomedicine. J. Nanobiotechnol..

[85] Cao M., Cai R., Zhao L., Guo M., Wang L., Wang Y., Zhang L., Wang X., Yao H., Xie C. (2021). Molybdenum derived from nanomaterials incorporates into molybdenum enzymes and affects their activities in vivo. Nat. Nanotechnol..

[86] Dogra P., Adolphi N.L., Wang Z., Lin Y.-S., Butler K.S., Durfee P.N., Croissant J.G., Noureddine A., Coker E.N., Bearer E.L. (2018). Establishing the effects of mesoporous silica nanoparticle properties on in vivo disposition using imaging-based pharmacokinetics. Nat. Commun..

[87] López-Estévez A.M., Lapuhs P., Pineiro-Alonso L., Alonso M.J. (2024). Personalized Cancer Nanomedicine: Overcoming Biological Barriers for Intracellular Delivery of Biopharmaceuticals. Adv. Mater..

[88] Saadh M.J., Mustafa M.A., Kumar A., Alamir H.T.A., Kumar A., Khudair S.A., Faisal A., Alubiady M.H.S., Jalal S.S., Shafik S.S. (2024). Stealth Nanocarriers in Cancer Therapy: A Comprehensive Review of Design, Functionality, and Clinical Applications. AAPS PharmSciTech.

[89] Desai N., Rana D., Patel M., Bajwa N., Prasad R., Vora L.K. (2025). Nanoparticle Therapeutics in Clinical Perspective: Classification, Marketed Products, and Regulatory Landscape. Small.

[90] Wong K.-Y., Nie Z., Wong M.-S., Wang Y., Liu J. (2024). Metal–Drug Coordination Nanoparticles and Hydrogels for Enhanced Delivery. Adv. Mater..

[91] Yan Z., Liu Z., Yang B., Zhu X., Song E., Song Y. (2023). Long-term exposure of molybdenum disulfide nanosheets leads to hepatic lipid accumulation and atherogenesis in apolipoprotein E deficient mice. NanoImpact.

[92] Qi Z., Yan Y., Xu Z., Chong W., Qiu Y., Huang X., Jing J., Fan H., Liang Q., Liu S. (2025). Molybdenum disulfide induces growth inhibition and autophagy-dependent hepatocyte cell death through directly binding and regulating the activity of MST2. Mater. Today Bio.

[93] Li J., Guiney L.M., Downing J.R., Wang X., Chang C.H., Jiang J., Liu Q., Liu X., Mei K.C., Liao Y.P. (2021). Dissolution of 2D Molybdenum Disulfide Generates Differential Toxicity among Liver Cell Types Compared to Non-Toxic 2D Boron Nitride Effects. Small.

[94] Lima A.F., Justo G.Z., Sousa A.A. (2024). Realizing active targeting in cancer nanomedicine with ultrasmall nanoparticles. Beilstein J. Nanotechnol..

[95] Long X., Wang J., Wang H., Hu K., Zhang W., Lin W., Fang C., Cheng K., Song Z. (2025). Injectable 2D-MoS_2_-integrated Bioadhesive Hydrogel as Photothermal-Derived and Drug-Delivery Implant for Colorectal Cancer Therapy. Adv. Healthc. Mater..

[96] Sun R., Chen H., Sutrisno L., Kawazoe N., Chen G. (2021). Nanomaterials and their composite scaffolds for photothermal therapy and tissue engineering applications. Sci. Technol. Adv. Mater..

[97] Wang X., Li T., Ma H., Zhai D., Jiang C., Chang J., Wang J., Wu C. (2017). A 3D-printed scaffold with MoS_2_ nanosheets for tumor therapy and tissue regeneration. NPG Asia Mater..

[98] Mülhopt S., Diabaté S., Dilger M., Adelhelm C., Anderlohr C., Bergfeldt T., Gómez de la Torre J., Jiang Y., Valsami-Jones E., Langevin D. (2018). Characterization of Nanoparticle Batch-To-Batch Variability. Nanomaterials.

[99] Faria M., Björnmalm M., Thurecht K.J., Kent S.J., Parton R.G., Kavallaris M., Johnston A.P.R., Gooding J.J., Corrie S.R., Boyd B.J. (2018). Minimum information reporting in bio–nano experimental literature. Nat. Nanotechnol..

[100] Sharifi S., Mahmoud N.N., Voke E., Landry M.P., Mahmoudi M. (2022). Importance of Standardizing Analytical Characterization Methodology for Improved Reliability of the Nanomedicine Literature. Nano-Micro Lett..

[101] Camacho Vieira C., Peltonen L., Karttunen A.P., Ribeiro A.J. (2024). Is it advantageous to use quality by design (QbD) to develop nanoparticle-based dosage forms for parenteral drug administration?. Int. J. Pharm..

[102] Nagpal S., Palaniappan T., Wang J.-W., Wacker M.G. (2024). Revisiting nanomedicine design strategies for follow-on products: A model-informed approach to optimize performance. J. Control. Release.

[103] Csóka I., Ismail R., Jójárt-Laczkovich O., Pallagi E. (2021). Regulatory Considerations, Challenges and Risk-based Approach in Nanomedicine Development. Curr. Med. Chem..

[104] Nag S., Mitra O., Tripathi G., Adur I., Mohanto S., Nama M., Samanta S., Gowda B.H.J., Subramaniyan V., Sundararajan V. (2024). Nanomaterials-assisted photothermal therapy for breast cancer: State-of-the-art advances and future perspectives. Photodiagn. Photodyn. Ther..

[105] Zhu L., Xu J., Ren J., Yang M., Yang C., Gao X., Bianco A., Ji D.K. (2025). A Biodegradable 2D Metallic MoS_2_ Genesheet for Synergistic NIR-II Photothermal Immunotherapy. Small.

[106] Younas A., Wang S., Asad M., Al Mamun A., Majeed S., Sharif A., Zhou Q., Liu Y., Geng P., Shao C. (2026). Recent advances in cancer nanomedicine: From smart targeting to personalized therapeutics—Pioneering a new era in precision oncology. Mater. Today Bio.

[107] U.S. Food and Drug Administration (2022). Drug Products, Including Biological Products, that Contain Nanomaterials—Guidance for Industry. https://www.fda.gov/regulatory-information/search-fda-guidance-documents/drug-products-including-biological-products-contain-nanomaterials-guidance-industry.

[108] Agency E.M. (2013). Reflection Paper on Surface Coatings: General Issues for Consideration Regarding Parenteral Administration of Coated Nanomedicine Products. https://www.ema.europa.eu/en/news/european-medicines-agency-publishes-reflection-paper-general-issues-consideration-regarding-coated-nanomedicines.

[109] Liu T., Chao Y., Gao M., Liang C., Chen Q., Song G., Cheng L., Liu Z. (2016). Ultra-small MoS2 nanodots with rapid body clearance for photothermal cancer therapy. Nano Res..

[110] Nieves L.M., Berkow E.K., Mossburg K.J., O N.H., Lau K.C., Rosario D.N., Singh P., Zhong X., Maidment A.D.A., Cormode D.P. (2024). Renally Excretable Molybdenum Disulfide Nanoparticles as Contrast Agents for Dual-Energy Mammography and Computed Tomography. Bioconjug. Chem..

